# Mobility patterns are associated with experienced income segregation in large US cities

**DOI:** 10.1038/s41467-021-24899-8

**Published:** 2021-07-30

**Authors:** Esteban Moro, Dan Calacci, Xiaowen Dong, Alex Pentland

**Affiliations:** 1 Media Lab, Massachusetts Institute of Technology, Cambridge, MA USA; 2 Departamento de Matemáticas & GISC, Universidad Carlos III de Madrid, Leganés, Spain; 3 Department of Engineering Science, University of Oxford, Oxford, UK

**Keywords:** Computational science, Social sciences

## Abstract

Traditional understanding of urban income segregation is largely based on static coarse-grained residential patterns. However, these do not capture the income segregation experience implied by the rich social interactions that happen in places that may relate to individual choices, opportunities, and mobility behavior. Using a large-scale high-resolution mobility data set of 4.5 million mobile phone users and 1.1 million places in 11 large American cities, we show that income segregation experienced in places and by individuals can differ greatly even within close spatial proximity. To further understand these fine-grained income segregation patterns, we introduce a Schelling extension of a well-known mobility model, and show that experienced income segregation is associated with an individual’s tendency to explore new places (place exploration) as well as places with visitors from different income groups (social exploration). Interestingly, while the latter is more strongly associated with demographic characteristics, the former is more strongly associated with mobility behavioral variables. Our results suggest that mobility behavior plays an important role in experienced income segregation of individuals. To measure this form of income segregation, urban researchers should take into account mobility behavior and not only residential patterns.

## Abstract (web summary)

Urban income segregation is often discussed in terms of where people live. Here, the authors show that the way people experience income segregation is also associated with their mobility patterns and the places they visit.

## Introduction

In a world of increasing urbanization, migration, and mobility, cities are becoming the epicenter of our social life. Diverse populations and social cohesion are crucial for sustainable urban development, but cities are facing rising segregation and inequality^1^. These two forces can erode the urban social fabric, dramatically affecting economic, social, and health outcomes of people living in urban areas. In particular, income segregation has been shown to impact access to important urban resources, such as housing^2^, community facilities^3^, health services^4^, and clean environment^5^. Recently, residential income segregation has been shown to have a significant effect on the economic outcome of children^6^.

To quantify income segregation, researchers often measure or approximate actual social interactions or exposure between different income groups in cities^7,8^. Because it is difficult to measure actual interactions between individuals in the real world, many studies instead quantify the potential opportunities people have to interact with others from different economic backgrounds. This is often measured as the amount of physical exposure to different income groups in one’s daily lives^9–11^, and income segregation is understood as a result of restrictions to contact with other groups^12^. However, most city-dwellers spend much of their time outside home^13,14^ and, interactions and encounters between people happen in specific places, not at the level of large neighborhoods or census areas. Income segregation actively experienced by people is indeed different from traditional measures of residential income segregation, and it varies by both the type of places visited and the time of the day^7,14–19^. Understanding the income segregation experience of individuals requires a more thorough understanding of behavior and mobility beyond residence, including individual motivation for visiting different places and encountering other groups of people.

The daily mobility of individuals in urban areas is now a well-studied subject. Research that uses call detail records or GPS locations at the city- and country-scale to measure high-resolution human movement has shown that individual mobility patterns are highly predictable^20–22^, explainable by urban mobility models^20,23–25^, and can be grouped into collective mobility behaviors^26^. These results suggest that experienced income segregation in the social fabric of cities might be partly encoded in universal behavior and mathematical models that explain the urban mobility patterns of people.

## Results

Using a large collection of micro-scale mobility data, we address how individual mobility behavior contributes to people’s experience of income segregation. Specifically, we analyze income segregation at the level of individual places in cities, and identify the main urban, behavioral, residential and mobility features associated with reduced or increased social connection and experienced income segregation in cities. Our main data source is from Cuebiq, who supplied 6-month long records of anonymized and high-resolution mobile location pings for 4.5 million devices across 11 U.S. census core-based statistical areas (CBSAs). Our second data source is a collection of ~1.1 million verified venues across all CBSAs, obtained via the Foursquare API (see Methods).

Each individual’s device in the data set is characterized by a corresponding socio-economic status (SES) proxy. We first infer the home area of each individual at the U.S. Census Block Group^27^ level using their most common location between 10 p.m. and 6 a.m. Individuals are then grouped in four equally sized quantiles of SES according to the median household income of their home area (see Methods Section and Supplementary Note 1.3). We further extract any visits an individual makes to a given place that lasts for more than 5 min. Several post-stratification techniques and comparison with other data sets were implemented to ensure the representativeness of the data at the level of population, income, and place attendance (see Methods section and Supplementary Note 1.4).

**Fig. 1:**
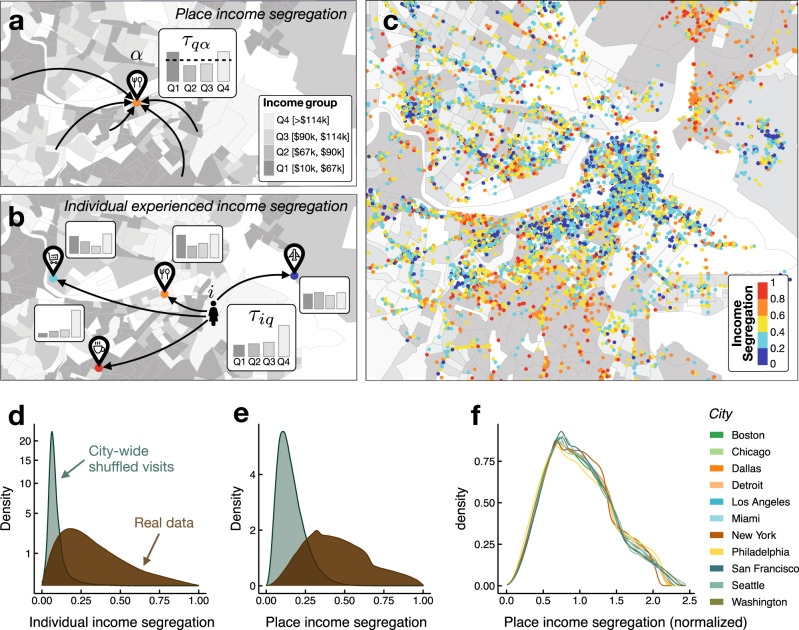
Place and individual income segregation. **a** People from different census block groups visit a given place. Using the median income of each census block group, we calculate the distribution of time spent by the four income groups (quartiles) in that place. Income segregation of the place measures the unevenness of this distribution [see Eq. (1)]. **b** For a given individual, we calculate the distribution of time that individual encounters people of different income groups in each place and in total. Individual income segregation measures the unevenness of this distribution, calculated similarly to Eq. (1). **c** Map of the places in downtown Boston color-coded according to their income segregation. **d**, **e** Distribution of individual/place income segregation values for all the cities (real data) compared with the one obtained by shuffling the visits of individuals at the city level. **f** Comparison of the distribution of place income segregation for different cities. For each city the values have been normalized by the average place segregation value for that city. Icons designed by bqlqn from flaticon.com. Maps were produced in R using the TIGER shapefiles from the U.S. Census Bureau^41^.

To measure the income segregation of each place *α* in the city, we compute the proportion of total time spent at that place by each income quartile (see Fig. 1a). We create a metric, *S*_*α*_, that quantifies income segregation as a measure between 0 and 1. A place is fully integrated (*S*_*α*_ = 0) when the total time across all individuals spent at the venue is split evenly among the four income quartiles. By contrast, a venue with *S*_*α*_ = 1 is one that is visited exclusively by a single income group, hence a higher level of segregation (see the Methods section). To define income segregation at the level of individual experience, we compute the amount of time they spend in each place and, using the place income segregation measure, calculate the relative exposure of an individual *i* to each income quartile *q* in the city (see Fig. 1b). We then construct a measure of experienced income segregation by individuals, *S*_*i*_, that mirrors our measure for places (see the Methods section). We have examined extensively that our results are robust against the specific choice of segregation metric or groups of income (see Supplementary Note 2.2).

### Income segregation of places

**Fig. 2:**
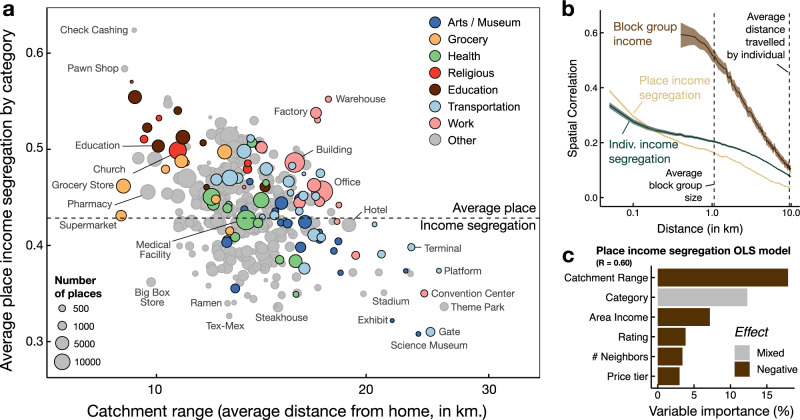
Different places have different income segregation. **a** Average place segregation by category as a function of the average distance traveled by individuals to reach that place from their home. Colors correspond to different groups of place categories and the size is proportional to the number of places in each category. As we can see the average income segregation depends both on the type of place and distance traveled. **b** Spatial correlation of the income segregation experienced at places and by individuals compared with that of census block group income. In the former cases, the correlation drops significantly at short distances, while income of census block groups are correlated even at distances larger than 1 km (vertical dashed line). **c** Summary of importance of each variables in the regression model for place segregation in Chicago (see Supplementary Table 3 for the results on the whole data set). Variables are color-coded according to their signed effect on income segregation and variable importance is measure according to the method in Lindeman et al.^42^.

Income segregation measured at the level of places is heterogeneous across places (see Fig. 1e); more importantly, it has a very granular spatial resolution: economically mixed places can be only a few dozen meters away from those that are highly segregated, even just across the street (see Fig. 1c for an illustration in downtown Boston). Figure 2b shows that while the spatial correlation of block group median income is quite high even for large distances (>10 km), place income segregation maintains a low level of correlation even locally (~50 m). Figure 1f shows the distribution of normalized place income segregation, *P*(*S*_*α*_), for each of the cities in our data set. Surprisingly, *P*(*S*_*α*_) is strikingly similar across a diverse array of US metro areas. These results show that the neighborhood or census area in which a place is located does not predict its income segregation profile: most areas in cities are home to both highly integrated and highly segregated places.

To understand the relationship between places and income segregation, we model the income segregation of each place in our data set using a simple regression model. We include variables that indicate a place’s rating, price tier, and category (Grocery Store, Convention Center, Office, Chinese Restaurant, etc). We also include the average distance users need to travel from their home census block group in order to visit a place, the number of other places in the immediate area, and the median income of the block group in which a place is located. Finally, to compensate for the difference between areas within the same city, we include geographical fixed effects at the level of Public Use Microdata Areas (PUMAs)^28^, which typically span about 20 km and contain a residential population of 150 thousand people. Figure 2c shows the importance of several variables in predicting a place’s income segregation (see Supplementary Note 5 for the details of the model). Apart from the PUMA in which a place is located, the two most important variables in predicting a place’s income segregation are its category and its average travel distance, which we refer to as *catchment range*. Place categories with lower catchment range (higher average travel distance to them) tend to be less segregated than categories that are highly accessible (Fig. 2a). Specifically, unique places in cities, such as arts venues, museums, and airports, tend to be highly integrated, while places that primarily serve local communities, such as places of worship and grocery stores, are generally more segregated by income. We interpret the latter to be an artifact of residential income segregation: people are more likely to visit grocery stores that are close to their home. Notice, however, that category and catchment range do not fully explain a place’s income segregation. Indeed, results in Fig. 2c suggest that they account only for 18 and 15% of the variance in place income segregation. Some categories in Fig. 2a (e.g., workplaces and restaurants) are dispersed in terms of both income segregation and catchment range. For example, Factories are much more segregated than Offices, Pawn Shops, and Supermarkets, despite similar catchment range. This result suggests that even people with the same mobility patterns might experience different levels of income segregation just by visiting different types of places.

### Income segregation experienced by individuals

The fine-grained structure of place income segregation and the fact that individuals move much longer distance than home census areas challenges the notion that experienced income segregation in cities is driven by residential neighborhoods or well-described by census areas. As shown in Fig. 2c, place income segregation depends only slightly on the income of the neighborhood in which a place is located. Visitors to a place also often do not live nearby: in our dataset individuals travel an average distance of 9.5 km to visit any given place. This suggests that most encounters in a city happen in places that are far beyond people’s home neighborhoods. On average, 78% of individuals’ encounters in our data are with people that live in another census PUMA region and, even more strikingly, only 3% of encounters happen between people that live in the same census block group. But what does this mean for income segregation experienced by individuals?

As we can see in Fig. 1d, experienced income segregation is quite heterogeneous across individuals. We find that individual experienced income segregation has a small correlation (*ρ* = − 0.173 ± 0.002) with the income of the area where the individual lives. Even individuals who live near one another can have very different income segregation experiences; as we can see in Fig. 2b, the spatial correlation of individual experienced income segregation drops significantly beyond 50 m. This suggests that individual experienced income segregation is not primarily described by where people live.

**Fig. 3:**
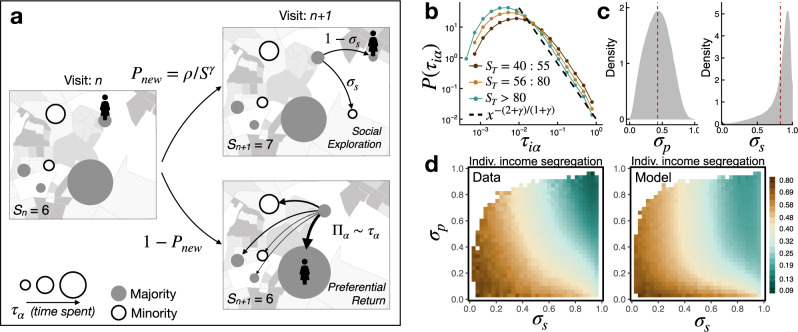
Social Exploration and Preferential Return (social-EPR) model. **a** Schematic description of the individual mobility model. After *n* stays the individual has visited *S*_*n*_ = 6 unique places. Those places are visited by majority (filled) or minority (empty) of their income group. Ball size is proportional to the fraction of time they spent at the place (*τ*_*α*_). For the *n* + 1 stay, the individual can either visit a new location with probability ${P}_{{{{{{{{\rm{new}}}}}}}}}=\rho /{S}_{n}^{\gamma }$ or returning to a previously visited location with probability 1 − *P*_new_. In the former case, the individual decides to visit a place where their group income is the majority with probability 1 − *σ*_*s*_, and explore other types of places with probability *σ*_*s*_. In the latter case, the next location will be chosen with probability Π_*α*_ ∝ *τ*_*α*_. **b** Distribution of the fraction of time spent at a place for different groups of *S*_*T*_. Dashed line corresponds to the analytical solution, i.e., $P({\tau }_{i\alpha }) \sim 1/{\tau }_{i\alpha }^{(2+\gamma )/(1+\gamma )}$, of the social-EPR model (see Supplementary Note 4). **c** Distributions of the observed values of place *σ*_*p*_ and social *σ*_*s*_ exploration for each individual in our data set. Dashed lines correspond to the mean of the distribution. **d** (Averaged) individual income segregation as a function of the place and social exploration for the real data and for the data produced by the social-EPR model.

Experienced income segregation for individuals is measured as the probability that an individual is exposed to different income groups in their daily mobility behavior, based on the income segregation patterns of the places they visit as well as the time they spend there (see Methods section). As is well known^20,23^, visitation patterns of individuals are rather uneven, and time spent in different places is heavy-tailed (see Fig. 3b). As a consequence, individuals spend most of their time in a small set of places (see more details in Supplementary Note 2.2). This suggests that for individuals, experienced income segregation is not driven by fleeting encounters in less visited places, but the places which are more consequential. In fact, if we calculate individual income segregation only using the top 10 places visited by any users, our measure is 97% correlated with that calculated using the whole set of places visited (see Supplementary Note 2.2). Even if the set of important places is on average small, similar to other works^29^, we find that some individuals (explorers) visit and spend time in many different places while others (returners) spend most of their time in few important locations. Thus the set of important places is larger for explorers than for returners. Remarkably, we find that this exploration/exploitation behavior emerges in many other cities and geographies and is correlated with experienced income segregation: if we denote *S*_*T*_ the total number of places visited, then returners (with a small *S*_*T*_) are found to be more segregated than explorers (with a large *S*_*T*_). Specifically, the correlation between experienced income segregation for individuals and the total number of places visited is high (*ρ* = −0.411 ± 0.001 after controlling for the number of visits, see Supplementary Note 4). This suggests that individual experienced income segregation is associated with the mobility visitation patterns within the city.

We also find that, in general, segregated individuals visit similarly segregated places. In principle, it could be possible that an integrated (nonsegregated) individual flits between segregated places that are dominated by different income groups. However, the correlation between individual experienced income segregation and the average income segregation of visited places is high (*ρ* = 0.579 ± 0.001), suggesting that individuals visit places of similar income segregation level to their own overall experience (see Supplementary Note 2.3). Furthermore, individuals are equally segregated by income between different types of places. For example, we find that individual income segregation calculated using only visits to more “social” places, such as Education, Colleges, Work places, Places of worship, Art/Museums, Sports or Entertainment venues, is correlated with the overall individual income segregation (*ρ* = 0.754 ± 0.001). This suggests that individuals tend to visit places with a given income segregation pattern throughout the city, and that there is a strong association of that pattern with the final experience of income segregation individuals have.

### Social EPR model

To better understand how place income segregation patterns is related to individual income segregation, we model individual mobility behavior using the well-known Exploration and Preferential Return (EPR) model^23^. The EPR model describe the visitation patterns of individuals in a city using two generic mechanisms: exploration (visiting a new place) and preferential return (visiting an already visited place) (see Fig. 3a). Using this model, an individual *i*’s mobility can be described by a single individual parameter *ρ*, which in turn can be sufficiently explained by an individual’s place exploration defined as *σ*_*p*_ = *S*_*T*_/*N*, where *S*_*T*_  ∝ *ρ* is the number of unique places *i* has visited and *N* is the total number of visits for *i* (see Supplementary Note 4). As we can see in Fig. 3c, users tend to visit new places frequently (average ${\overline{\sigma }}_{p}$ = 0.43), but there also exists a large fraction of explorers (${\sigma }_{p} \; > \; {\overline{\sigma }}_{p}$) and returners (${\sigma }_{p} \; < \; {\overline{\sigma }}_{p}$). We find that the EPR model accurately explains patterns of individual visits to different places; for example, the distribution of time by place is highly uneven and follows the Zip’s law $P({\tau }_{i\alpha }) \sim {\tau }_{i\alpha }^{-\beta }$ (see Fig. 3b and Supplementary Note 4). We also find that, as expected, explorers spend less time in the top places than returners. However, the model does not explain the variability we observe in experienced income segregation of individuals. Indeed, the EPR model assumes that the places an individual visits are chosen randomly throughout the city^23^, or only within local areas, independently of how segregated the place is. This assumption implies that all individuals from a given area would have very similar experience of income segregation, which empirically is not the case.

To rectify this, we extend the EPR model to account for the income segregation patterns of the places visited by individuals. To this end, we introduce a parameter, reminiscent of that in the segregation model proposed by Thomas Schelling^30^, which quantifies whether the majority of a place’s visitors are from the same income group as the user. More specifically, we characterize each individual by their Schelling parameter or social exploration rate, *σ*_*s*_, which is defined as their probability of visiting a new place where their income group is a minority (see Methods section and Supplementary Note 4). In other words, *σ*_*s*_ is the fraction of unique places visited where their income group is a minority. The parameter *σ*_*s*_ describes the income segregation patterns of the places visited by an individual, but in a particular way: individuals with small *σ*_*s*_ spend most of their time in places in which their income groups are the majority. Therefore, not only are those places segregated by income, but also they are towards those individuals’ income group. On the other hand, we are making the assumption that *σ*_*s*_ predicts the choice of places independently of the time spent there. Our data corroborate this assumption: the correlation between the income segregation of places and the time spent there is small (*ρ*[*S*_*α*_, *τ*_*iα*_] = 0.049 ± 0.001). However, we observed in general that top places are slightly (~14%) more segregated than the rest (see Supplementary Note 4 for further information).

This modified social-EPR model, with only two parameters *σ*_*p*_ and *σ*_*s*_, well explains the general visitation patterns of and income segregation experienced by an individual: income segregation measures produced by the model are correlated (*ρ* = 0.777 ± 0.001) with observed ones (see Fig. 3d and Supplementary Note 4). Thus, a generative model based on just two parameters can accurately explain the variability in experienced income segregation for 1.03 million individuals in 11 different cities in the US.

Note that individual experienced income segregation is not explained merely by *σ*_*s*_: although individuals with a very small *σ*_*s*_ would be largely segregated, the majority of people in our data set have a large *σ*_*s*_ (~80% of them have *σ*_*s*_ > 0.75, see Fig. 3c). For the latter group, it is the interplay between *σ*_*p*_ and *σ*_*s*_ that predicts an individual’s overall level of experienced income segregation. The reason for this is that, while *σ*_*s*_ controls the segregated nature of the places a person visits, *σ*_*p*_ describes how often (or not) they are visited. People with a small *σ*_*p*_ (returners) spend most of their time in a small number of places that are likely near their neighborhoods, and their experienced income segregation is primarily predicted by how segregated those places are. Since top places are slightly (~14%) more segregated than the rest, this means that in general returners are more segregated than explorers, even if their *σ*_*s*_ is large. Only people with both large *σ*_*s*_ and *σ*_*p*_, who have high rates of both social exploration and place exploration, are economically integrated and have equal exposure to all the income groups in the city. Indeed, it is important to note that individual experienced income segregation is not just driven by *σ*_*s*_: while the results of our social-EPR model are correlated with the empirical data (*ρ* = 0.777 ± 0.001), the correlation between individual income segregation and *σ*_*s*_ is only moderate (*ρ* = −0.538 ± 0.002). Finally, both aspects of exploration seem to be largely independent from each other with only a moderate correlation (*ρ*[*σ*_*p*_, *σ*_*s*_] = 0.126 ± 0.002). These results reinforce the idea that experienced individual income segregation depends on both the visitation patterns and the income segregation of the places where individuals spend their time. The only way to be economically integrated is to be a social and place explorer at the same time.

### Explaining exploration and experienced income segregation

The proposed social-EPR model suggests that experienced income segregation for individuals is largely described by two characteristics: social exploration (measured by *σ*_*s*_), and place exploration (measured by *σ*_*p*_). What predicts an individual’s social and place exploration patterns? Traditional ways of understanding income segregation have tried to answer this question by looking at the different demographic characteristics of residential neighborhoods or workplaces. However, as we have already seen, the way we move around the city and the places we visit are important factors associated with individual experienced income segregation.

**Fig. 4:**
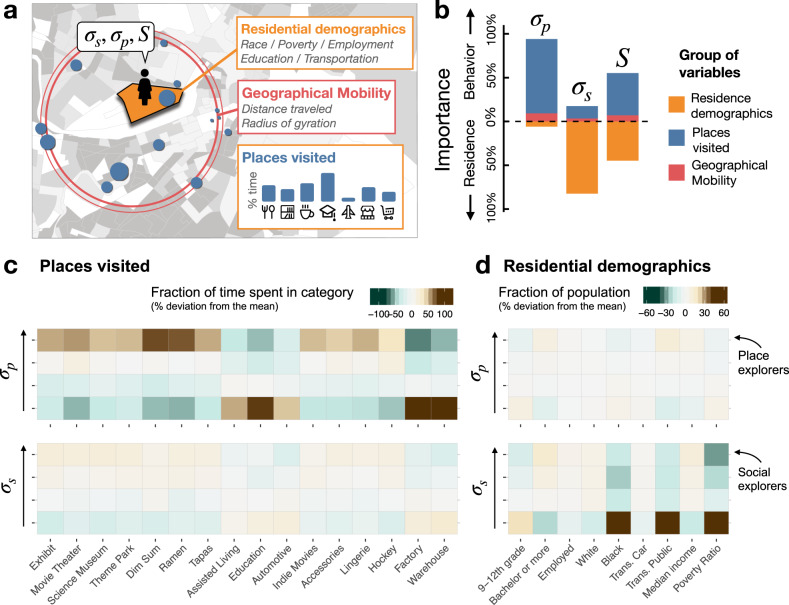
What explains place and social exploration?. **a** To explain social (*σ*_*s*_) and place (*σ*_*p*_) exploration, as well as experienced income segregation (*S*), for each individual, we constructed a number of demographic variables from their residence (at the level of census block group), variables about the amount of time they spent in each place category, and variables about their geographical mobility. **b** Percentage of variance explained by different groups of variables (Residence, Places or Geographical Mobility), estimated via regression models for *σ*_*p*_, *σ*_*s*_ and *S*. **c** Relative fraction of time spent by individuals in different groups (quantiles) of place exploration (top) and social exploration (bottom). Individuals with high place exploration are more likely to visit categories related to entertainment, food, and shopping, while those with low place exploration visit Education, Factories, and Warehouses. This behavior is not found for social exploration (*σ*_*s*_), where all categories are visited more or less evenly by different groups. **d** Residential demographic characteristics for the different groups of place exploration (top) and social exploration (bottom). Social exploration is correlated with variables such a Poverty, use of Public Transportation, or percentage of white/Black population. However, place exploration is almost independent of those demographic characteristics.

To understand the relative importance of traditional and mobility behavioral factors in individual experienced income segregation, we investigate three dimensions that could affect the types of encounters an individual may have in a city (see Fig. 4a): (1) lifestyles, or mobility behavioral variables, i.e., the places a person visits in a city, which provide different social and economic choices and opportunities, and have different income segregation profiles; (2) geographical mobility, i.e., the extent of the city covered in their daily mobility; and (3) residence, i.e., demographic characteristics of a users’ residential neighborhood. The relative weight of those dimensions is investigated using a simple linear regression, which models the individual parameters *σ*_*p*_ and *σ*_*s*_ as well as the individual experienced income segregation *S*_*i*_ as a function of behavioral (lifestyles + geographical mobility) and residential variables (see Supplementary Note 6, and Supplementary Tables 4, 5 and 6 for more details). Note that the model includes fixed variables (PUMA) to account for the area in which individuals live, as well as variable related to an individual’s geographical mobility. By doing so, the model accounts for the extent of the area covered by individuals in the city and potential heterogeneity in the opportunity structure around the city. This allows us to investigate the effect of individual opportunity (implied by lifestyles), as opposed to structural (geographical) opportunity, on income segregation experience.

As we can see in Fig. 4b, different groups of variables are related to different dimensions of the social-EPR model. The place exploration parameter *σ*_*p*_ is mostly influenced by mobility behavioral variables, i.e., types of places an individual visits and in a minor way by their geographical mobility. This result is in line with recent studies^14,31^ which report insignificant relationship between place exploration behavior and socio-economic variables. Our results indicate that place explorers can be found in any area, mostly independent of their demographics, and that the key ingredient to understand place exploration is the lifestyles of individuals. For example, knowing that an individual frequently visits Movie Theaters, Exhibits, Coffee Shops or certain types of Restaurants (e.g., Tapas, Dim Sum, Ramen) can tell us more about whether an individual is a place explorer (high *σ*_*p*_) than knowing the income level of the individual (see Fig. 4c), despite that the time spent on those categories is insignificant (only 1.2% of time is spent on Coffee Shops, and 0.3% in Theaters) and that those visits do not affect directly the individual’s experienced income segregation. In general, individuals that have high place exploration (*σ*_*p*_) are those that have lifestyles that include visits to Entertainment, Food and Shopping places, but not many visits to places associated with Education and certain Work places like Factory or Warehouse (see Fig. 4c and Supplementary Table 6).

On the other hand, we find that the social exploration parameter *σ*_*s*_ is mostly (82% of the variance explained) predicted by the residential characteristics of a user’s neighborhood, especially education level, employment, race composition, mode of transportation, and poverty level (see Fig. 4d). Residents of neighborhoods with higher education level, higher median income, less Black residents, less usage of public transportation, and lower poverty ratio are more likely to have a higher *σ*_*s*_, and visit new venues across the city where they are a minority. Interestingly, these results are similar to survey results on residential preferences^32,33^.

We also find that lifestyles of individuals play a minor role on *σ*_*s*_ (see Fig. 4b). The type of places visited only accounts for about 14% of its variance. Of course, people who spend most of their time only at segregated places such as Education, local Groceries or Pawn Shops will have smaller *σ*_*s*_ because those places tend to be segregated by income (see Fig. 2 and Supplementary Table 5). But this group of people are a small minority. In general, most people spend the majority (~46%) of their time in place categories such as Work, Food and Service places, which have many different, both economically segregated and integrated, places across the city (see Supplementary Note 7). This means that even people with the same lifestyles can have very different *σ*_*s*_. As a result, *σ*_*s*_ depends only slightly on the type of places visited (see Fig. 4c), suggesting that there are no particular institutions in the city which affect or reveal the social exploration nature of individuals. Only demographic features that are also explicitly expressed in residential preferences are related to whether people are social explorers or not.

Our results demonstrate that *σ*_*p*_ and *σ*_*s*_ measure different, almost independent aspects of experienced income segregation in urban environment. Place exploration—how often an individual explores new places in physical space—is related mostly to an individual’s lifestyles, which depend on the type and accessibility of places they visit in their daily lives, while social exploration—how likely people are to visit places with visitors that are different from themselves—seems to be embedded in demographic and residential characteristics. As a result, both residential and mobility behavioral features play an important role in explaining the overall individual income segregation: in the model for *S*_*i*_, mobility behavioral factors, and in particular the types of places individuals visit, account for 55% of relative importance, while residential (census) factors account for the remaining 45% (see Fig. 4b). This is an important result suggesting that how people experience economic inequality and income segregation in their daily lives is heavily dependent on both mobility behavioral patterns as well as where they live. For example, people living in high income neighborhoods have in general larger social exploration, but that does not always translate into larger place exploration. Hence, high income people can be as segregated as low income people (see Fig. 3c). On the contrary, people living in deprived areas can be integrated if they are place explorers. As we mentioned before, only people who are both social and place explorers are economically integrated.

## Discussion

Two thirds of the world’s population will live in cities by 2050 according to the United Nations. As a consequence, urban income segregation is an increasingly critical issue that challenges societies across the globe^1^. In this context, our study includes two important contributions towards understanding income segregation in cities, understood as restrictions to interaction with other groups^6,9,12,14,15,18^. First, our approach frames experienced income segregation in cities as a behavioral process that emerges at the level of places, rather than a static attribute at the level of regions or neighborhoods^13^. A place’s category has a strong relationship with how economically segregated it is, and this relationship is consistent between different cities in the US. Second, by modeling income segregation as a behavioral process, we show that how people experience income segregation in cities is heavily associated with two attributes: their tendency to seek out new places to visit, and how often they find themselves a minority in the new places they explore. These two types of exploration are strongly related to an individual’s mobility behavioral patterns and residential characteristics, respectively, which shows that experienced income segregation is associated with not only where people lives, but also their visitation patterns which might reflect the opportunities available to them and choices they make.

We demonstrate that a simple Schelling extension to a classic model of individual mobility can be used to accurately model income segregation experienced by individuals. The proposed social-EPR model provides a bridge between computational studies of human mobility^20,22–25^ and the income segregation literature^30,33^, and reveals the importance of place and social exploration on economic integration in cities. We believe that the simplicity of the social-EPR model and the way we operationalize the measurement of social and place exploration provides a conceptual framework for future studies that can further explore what residential or behavioral factors might increase people’s exploration in physical or social space. More importantly, our results suggest that other processes that heavily depend on urban mobility (from transportation to environmental pollution or epidemics) might also depend on individual social and place exploration. Our model thus provides a new venue to understand the nuances of how income segregation is experienced in cities, and may provide further opportunities to investigate its relation to other important aspects of city life.

Our findings have implications for our understanding of how income segregation is experienced in cities. While income segregation is often studied in terms of neighborhoods, areas, and residences, our results show that the income segregation experienced by individuals is related to their visitation patterns and mobility behaviors. To better understand how income segregation manifests itself in cities, understanding of residential segregation should be complemented with how urban interventions and design impact both social and place exploration, i.e., where residents spend their time well beyond their neighborhoods, and with diverse groups of people. For example, transit routes, commercial development strategies and zoning, and prioritizing certain types of amenities may all affect the lifestyles of city residents, at which venues they may spend time, and by extension, with whom they have the opportunity to encounter. Our results suggest how further analysis of those interventions (or natural experiments) may help understand the causal effect on experienced income segregation. Since spatial proximity still plays an important role in creating social relationships^34^, alleviating experienced income segregation may help create more diverse and robust societies in the future.

Our study has several limitations. We select individuals for whom we could identify home locations during the 6-month period, and therefore exclude those who do not have a stable residence or have nonnormative work shifts (i.e., between 8 p.m. and 4 a.m.). Similarly, the venues we consider are limited to those available via the Foursquare API, which might be biased towards certain types of places. We are also not able to differentiate between encounters of different nature, e.g., a casual conversation between two strangers in a coffee shop, or a financial transaction between a service worker and a high-finance banker^35^. Our results therefore serve as a proxy and bound for the potential income segregation in cities. Our study focuses on income (economic) segregation and not segregation in other dimensions such as race, wealth, or ethnicity, which might be correlated but nevertheless different from income segregation. Finally, although our results are descriptive and do not imply causal relations, we believe that our findings point to important factors whose causal effect may be further tested through carefully designed experiments and interventions.

## Methods

### Mobility data

Our geo-location data come from Cuebiq, a location intelligence company that curates, creates and analyzes high-resolution location data from applications of opted-in users in an anonymized way. The data set consists of anonymized records of GPS locations (“pings”) from users that opted-in to share the data anonymously through a General Data Protection Regulation and California Consumer Privacy Act compliant framework. Data was shared in 2017 under a strict contract with Cuebiq through their Data for Good program where they provide access to de-identified and privacy-enhanced mobility data for academic research and humanitarian initiatives only. All researchers were contractually obligated not to share data further or attempt to de-identify data. Ethical oversight: Additionally, we obtained IRB exemption to use the mobility data from the MIT IRB office through protocols #1812635835 and its extension #E-2962.

We only consider pings which happen within 11 CBSA^36^ between Oct 2016 and March 2017 (see Table 1). We considered CBSAs instead of other geographical units, since they are areas that are socially and economically related to an urban center. This provides a self-contained metropolitan area in which people move for work, leisure or other activities. Note that most of the CBSAs we consider span several states. The metropolitan areas included in the study are (short names in parenthesis): New York-Jersey City (New York), Los Angeles-Long Beach-Anaheim (Los Angeles), Chicago-Naperfille-Elgin (Chicago), Dallas-Fort Worth-Arlington (Dallas), Philadelphia-Camden-Wilmington (Philadelphia), Washington-Arlington-Alexandria (Washington), Miami-Fort Lauderdale-West Palm Beach (Miami), Boston-Cambridge-Newton (Boston), San Francisco-Oakland-Hayward (San Francisco), Detroit-Warren-Dearborn (Detroit), and Seattle-Tacoma-Bellevue (Seattle), see Table 1. The initial data consisted of 70.2 billion pings from 14.3 million of unique smartphones. To control for smartphones that appear in our CBSAs for only short periods of time, we only consider devices with more than 2000 pings, giving us a filtered data set of 67.0 billion pings from 4.5 million unique smartphones.

**Table 1:** Description of each of the core-based statistical areas considered and some statistics about our data set.

CBSA name	State	Population	# smartphones	# stays
New York-Jersey City	NY-NJ-PA	19.83 M	558 k	141 M
Los Angeles-Long Beach-Anaheim	CA	13.05 M	630 k	120 M
Chicago-Naperfille-Elgin	IL-IN-WI	9.52 M	411 k	123 M
Dallas-Fort Worth-Arlington	TX	6.70 M	447 k	105 M
Philadelphia-Camden-Wilmington	PA-NJ-DE-MD	6.02 M	432 k	113 M
Washington-Arlington-Alexandria	DC-VA-MD-WV	5.86 M	212 k	55 M
Miami-Fort Lauderdale-West Palm Beach	FL	5.76 M	270 k	74 M
Boston-Cambridge-Newton	MA-NH	4.64 M	123 k	30 M
San Francisco-Oakland-Hayward	CA	4.46 M	504 k	129 M
Detroit-Warren-Dearborn	MI	4.29 M	338 k	105 M
Seattle-Tacoma-Bellevue	WA	3.55 M	100 k	28 M
Total		83.5 M	3.6 M	976 M

### Home and visits extraction

To assign individuals as having visited a place, we first extract stays from the raw location trajectories using the Hariharan and Toyama algorithm^37^, producing a data set of clustered locations, times, and duration of stays for each individual. We then perform a nearest-neighbor search to find the closest venue to each cluster. We discard stays that last for under 5 min or over 1 day, and clusters that do not have a venue within a 200-m radius. See Supplementary Note 1 for further details about our method to extract stays and attribute visits and different sensitivity tests of our results to our method. To characterize individuals in the data set with a corresponding income measure, we infer the home area of each individual at the Census Block Group level as measured by the 2012–2016 5-year American Community Survey (ACS) using its most common location between the hours of 10:00 p.m. and 6:00 a.m. We then use that block group’s median household income as a proxy for the anonymous phone individual’s income. We further discard any individuals that we identify as spending fewer than 10 nights in their home Census Block Group over the observation period, leaving us with a final data set of 976 million stays from 3.6 million anonymous individuals. Calculation of experienced income segregation is only done for 1.9 million anonymous individuals who have visits to our set of venues. Post-stratification techniques^38^ were implemented to assure the representativeness of the data at the level of population, income and place attendance. For the most active places we also found similar results using geo-localized data sets from Twitter and official attendance to large events. See Supplementary Notes 1.4 and 1.4.4 for further details.

### Other data

Demographic data at the level of Census Block group was obtained from the 2012–2016 5-year ACS^27^. Venues’ location and category were obtained via Foursquare using their Public Search API in 2017 and according to their terms and conditions of use. In our analysis, we only considered the ~1 Million venues that are visited by more than 20 unique anonymous individuals in our dataset. Venue categories follow the Foursquare classification^39^ but we also grouped manually the venues in our own Taxonomy of 13 groups, Art/Museum, City/Outdoors, Coffee/Tea, College, Entertainment, Food, Grocery, Health, Religious, Education, Service, Shopping, Sports, Transportation, Work. See Supplementary Table 2 and Supplementary Note 7 for further details about this Taxonomy.

### Measuring income segregation of places

To measure the income segregation of each place *α* in the city, we compute the proportion of total time spent at that place *α* by each income quartile *q*, *τ*_*qα*_, defining separate quartiles for each city. We define full integration of a place as *τ*_*qα*_ = 1/4 for each *q*, that is, the total time spent at venue *α* is split evenly across our four income quartiles. We then define the income segregation for each place *α*, *S*_*α*_, as any deviation from our idealized measure of integration:

1 $${S}_{\alpha }=\frac{2}{3}\mathop{\sum}\limits_{q}\left|{\tau }_{q\alpha }-\frac{1}{4}\right|.$$

The measure *S*_*α*_ is bounded between 0 and 1. A place with *S*_*α*_ = 0 means that a venue *α* is visited equally by all income quartiles in the city, with no deviation from our idealized integration measure of *τ*_*qα*_ = 1/4. By contrast, a venue with *S*_*α*_ = 1 is one that is visited exclusively by a single income group. Therefore, a higher *S*_*α*_ measure indicates that a place is visited more exclusively by a single income group, hence a higher level of income segregation. Our metric of segregation is similar to other typical segregation measures like the entropy or interaction coefficient within a place^40^, and our results are robust to changes in how segregation is defined (see Supplementary Note 3). Note that because our income groups are defined by population quartiles, *S*_*α*_ is defined relative to the actual household income distribution in each CBSA.

### Measuring income segregation experienced by individuals

If *τ*_*iα*_ is the proportion of time individual *i* has spent at place *α*, then we can define a individual’s relative exposure to income quartile *q*, *τ*_*iq*_, as a sum over all places *α* visited by individual *i*: *τ*_*iq*_ = ∑_*α*_*τ*_*iα*_*τ*_*qα*_, where *τ*_*qα*_ represents the proportion of time at place *α* spent by income group *q*. This effectively represents the probability that an individual is exposed to income group *q* in their daily behavior. Using this measure, we can then define individual income segregation, *S*_*i*_, as a simple rewriting of Eq. (1): ${S}_{i}=\frac{2}{3}{\sum }_{q}\left|{\tau }_{iq}-\frac{1}{4}\right|$. Our metric for individual income segregation can be thought of as an extension of the traditional metric of isolation or interaction for groups to the level of individuals based on daily encounters among them. While the mobility data set we use is large, co-location events between individuals are still quite sparse. Because of this sparsity, and to protect individual privacy in our analysis, we adopt this probabilistic approach to measuring encounters (see Supplementary Note 6). This choice does not change our main findings, and provides more statistical robustness to our measures.

### Social EPR model

In the EPR model each time an individual visits a place, it is a new one with probability *P*_new_, or the individual returns to a previous visited place with probability 1 − *P*_new_. According to the EPR model, ${P}_{{{{{{{{\rm{new}}}}}}}}}=\rho {S}_{n}^{-\gamma }$, where *S*_*n*_ is the number of unique places the individual has visited up until visit *n*. For places that have already been visited, the probability that an individual *i* visits a place *α*, Π_*α*_, is proportional to the amount of time that individual has spent there in the past, *τ*_*iα*_. We validate these hypotheses and find that, when we fit the model’s parameters to our data, we obtain *γ* ≃ 0.23 ± 0.02 which is similar to what has been reported in a few other studies of urban mobility^23^ (see Supplementary Note 4). The information contained in *ρ* can be equivalently captured by an individual *i*’s place exploration, which we define as *σ*_*p*_ = *S*_*T*_/*N*, where *S*_*T*_ is the number of unique places *i* has visited and *N* is the total number of stays for *i* (see Supplementary Note 4).

The proposed social-EPR model is an extension of the EPR model with an additional parameter. Specifically, when individuals decide to explore a new place (as in the EPR model), with probability *σ*_*s*_ they explore a new place where their income group is the minority. The choice of 50% (majority) in the Schelling model is not arbitrary. As we show in section Supplementary Note 4, we chose 50% because the social-EPR model is optimal around that value in the sense that the correlation between the model and the empirical data is maximum. The social-EPR model thus contains two parameters: *σ*_*p*_ and *σ*_*s*_. While *σ*_*s*_ controls the segregated nature of the places a person visits, *σ*_*p*_ describes how often (or not) they are visited.

### Regression models

To understand the relative importance of each group of variables and maintain explainability, we use a simple linear regression model *S*, *σ* ~ {*R*_*i*_} + {P_*i*_} + {*M*_*i*_} (see Supplementary Note 6 and Supplementary Tables 4, 5, and 6), where we model the individual parameters *σ*_*p*_ and *σ*_*s*_ as a function of mobility variables {*M*_*i*_}, lifestyles or type of places {P_*i*_}) and residential variables {*R*_*i*_}. Mobility variables are related to the extent of the city covered by individuals, i.e., radius of gyration and total distance traveled. Lifestyle variables are given by a vector {P_*i*_} whose entries correspond to the fraction of time user *i* has spent in each of the categories included in Fig. 2. Finally, we construct a vector {*R*_*i*_} of about 30 residential (census) variables that account for the income, education levels, employment characteristics, race composition, poverty ratios, transportation modes, etc. of each census block group (see Supplementary Table 1). See Supplementary Note 6 for a complete list of variables included in each group.

### Reporting summary

Further information on research design is available in the Nature Research Reporting Summary linked to this article.

## Supplementary information

Supplementary Information

Reporting Summary

## Data Availability

The data that support the findings of this study are available from Cuebiq through their Data for Good program, but restrictions apply to the availability of these data, which were used under the licence for the current study and are therefore not publicly available. Information about how to request access to the data and its conditions and limitations can be found in https://www.cuebiq.com/about/data-for-good/. Venues location and category were obtained via Foursquare using their Public Search API. Source anonymized aggregated data to reproduce our results are provided with this paper and are publicly available on github: https://github.com/emoro/Mobility_income_segregation. Source data are provided with this paper.

## Source data

Source Data

## Code availability

The analysis was conducted using the R statistical software system. Code to reproduce our main results in the figures from the aggregated data is publicly available on github https://github.com/emoro/Mobility_income_segregation.
