# Supplementary material for: Mobility patterns are associated with experienced income segregation in large US cities
Source: Nat Commun. 2021 Jul 30;12:4633. doi: 10.1038/s41467-021-24899-8 (PMC8324796; doi:10.1038/s41467-021-24899-8)
Supplement: Supplementary file 1 — Supplementary Information [file 41467_2021_24899_MOESM1_ESM.pdf]

Supplementary Information for *Mobility patterns are associated with experienced income segregation in large US cities*

Esteban Moro[1,2,*], Dan Calacci[1], Xiaowen Dong[1,3], & Alex Pentland[1]

[1]Media Laboratory, Massachusetts Institute of Technology, Cambridge, MA 02139, USA
[2]Departamento de Matemáticas & GISC, Universidad Carlos III de Madrid, 28911 Leganés, Spain
[3]Department of Engineering Science, University of Oxford, Oxford, UK
[*]To whom correspondence should be addressed; E-mail: esteban.moroegido@gmail.com

June 29, 2021

Supplementary Notes

1 Location data **3**
1.1 Extracting stays . . . 3
1.2 Attribution of stays to places . . . 3
1.3 Identifying home and economic status . . . 4
1.4 Representativeness of the data . . . 5
1.4.1 Population representativeness . . . 5
1.4.2 Income representativeness . . . 6
1.4.3 Comparing official attendance to professional sports games . . . 7
1.4.4 Other datasets . . . 8

2 Measuring Place and Individual Income segregation **8**
2.1 Place income segregation . . . 8
2.2 Individual experienced income segregation . . . 9
2.3 Relationship between individual and place income segregation . . . 11

3 Other measures of income segregation **12**

4 The social exploration and preferential return (social-EPR) model **12**
4.1 Definition of the model . . . 12
4.2 Testing the hypothesis of the model . . . 13
4.3 Measuring the parameters of the social-EPR model . . . 13
4.4 Simulating the social-EPR model . . . 14

5 Model for Place Income Segregation **16**

6 Model for Individual Experienced Income Segregation **16**
6.1 Independence of residential and places variables . . . 17

7 Venue categories **17**

8 Software used **18**

List of Supplementary Figures

1 Illustration of the Hariharan & Toyama algorithm to detect stays 3
2 Income groups for the cities considered 4
3 Correlation between the smartphone population detected in our data and Census population 6
4 Comparison of our data with income groups and official attendance to professional sport games 7
5 Comparison of the average income segregation by place category with Twitter data 8
6 Comparison between our probabilistic approximation for co-locations and real co-locations . 10
7 Comparison between individual income segregation calculated with all or just top N most visited places 10
8 Comparison between individual income segregation and the average income segregation of the places visited by the individual 11
9 Comparison of our income segregation metric with other inequality metrics 13
10 Testing the hypothesis and results of the EPR model 15
11 Dependence on the Schelling threshold 15
12 Time and dependence on income segregation of different type of places 18

List of Supplementary Tables

1 Summary statistics of the 29 Census variables used 19
2 Summary statistics of the venues categories used in our models grouped by types of places. . 20
3 Regression table for the place income segregation model in each metro area 28
4 Regression table for the different models of individual income segregation S_i 29
5 Regression table for the different models of individual social exploration S_i 30
6 Regression table for the different models of individual place exploration $\sigma_{p,i}$ 31

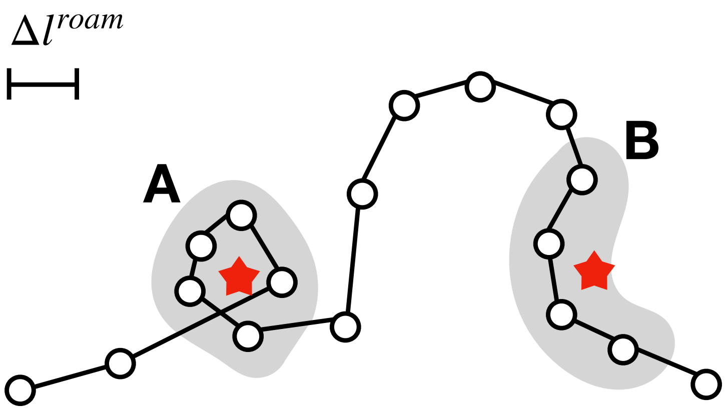

Supplementary Figure 1: Illustration of the Hariharan & Toyama algorithm [1] to detect stays. Each point corresponds to a ping for a given user; lines are drawn between consecutive points. Cluster A of pings is identified as a stay since the maximum distance of the pings in the cluster to their centroid (red star) is smaller than Δl^{roam}. However, cluster B of pings is not a stay.

1 Location data

1.1 Extracting stays

Since we are interested in detecting when users are exposed to others, we extracted *stays* from the individual trajectories, i.e., sequences of pings made when users spend some time in a single place. Those sequences of pings are spatially limited to small regions, and to detect them we use the algorithm proposed by Hariharan and Toyama [1], in which a set of consecutive pings are clustered together if their maximum distance from their centroid is not larger than some roaming distance Δl^{roam} (see Supplementary Figure 1). The duration of a stay, Δt^{dur}, is then the time between the first and last ping belonging to a cluster. Here, we use $\Delta l^{roam} = 50$ meters and we only use stays for which $5\text{min} \leq \Delta t^{dur} \leq 1\text{day}$. After using this algorithm and discarding users who do not have any stays, we are left with a total of 1.1 billion stays in 11 CBSAs.

1.2 Attribution of stays to places

A cornerstone of our analysis is based on modeling the attribution of stays to places. Like stays, each place is also represented by a single point in space. To attribute a stay to a place, we simply attribute each stay to the closest place in our dataset. Despite its simplicity, this strategy works well for inferring visited places from sparse, low accuracy mobile data [2]. To avoid attributing a stay to a distant place, we choose only the closest place within a radius of $d_{\max} = 100$ meters. If a stay is further than $d_{\max}$ from any venue, we do not attribute it to any place. Although we use $d_{\max} = 100$ meters, the average distance of a stay to the (closest) attributed venue is around 26 meters.

We have also tested the robustness of our results for different values of $d_{\max}$. We find that the correlation between place and individual income segregation using different values of $d_{\max}$ is very high: in particular for places we get $\rho[S_\alpha^{d_{\max}=100m}, S_\alpha^{d_{\max}=50m}] = 0.950 \pm 0.001$ and $\rho[S_\alpha^{d_{\max}=100m}, S_\alpha^{d_{\max}=150m}] = 0.972 \pm 0.001$, while for individual experienced income segregation $\rho[S_i^{d_{\max}=100m}, S_i^{d_{\max}=50m}] = 0.840 \pm 0.001$ and $\rho[S_i^{d_{\max}=100m}, S_i^{d_{\max}=150m}] = 0.918 \pm 0.001$. This test shows that our values for individual and place income segregation are largely independent of the details of our attribution algorithm.

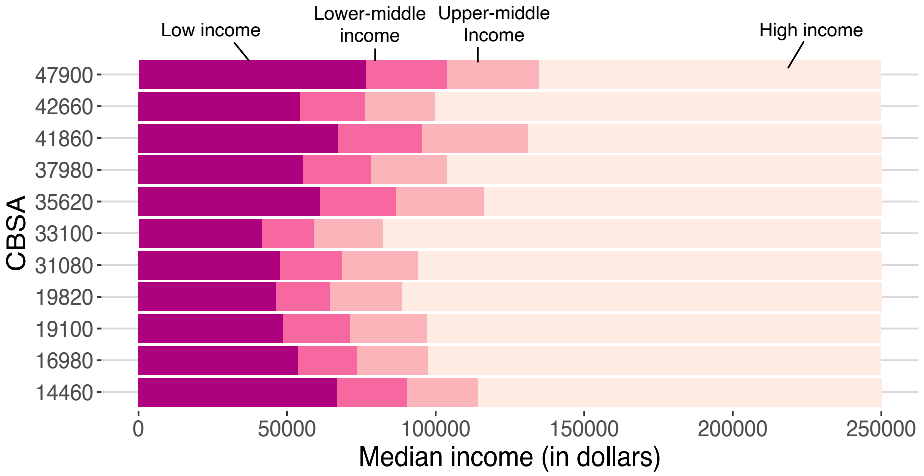

Supplementary Figure 2: Definition of the different income groups for the cities considered. By definition, each group contains 25% of the population in each city, but their income intervals are different. Source data are provided as a Source Data file.

1.3 Identifying home and economic status

For each smartphone, we use its stays from 22:00 to 06:00 and spatially cluster them using the Density-based Spatial Clustering of Applications with Boise (DBSCAN) algorithm [3] to detect the most likely cluster of stays each individual is located in during nighttime and early morning hours. We use 5 as the minimum number of points per cluster and $\varepsilon = 30$ meters as the neighborhood distance. We only consider individuals who were at their home cluster at least 10 nights in the observation period, which leads us to consider only 3.6 million individuals and 976 million stays (see Table 1 in the main text). Calculation of experienced income segregation is only done for 1.9 million anonymous individuals who have visits to our set of venues.

We estimate each individual's economic status using the income of the Census Block Group (CBG) where their home cluster is located. Census block groups are a geographical unit used by the United States Census Bureau and it is the second smallest geographical unit for which the bureau publishes data. Typically, CBG have a population of 600 to 3,000 people and in our CBSAs there are around 57 thousand of them. Using the 2012-2016 5-year American Community Survey (ACS) [4], we used the median household income in each census block group as a proxy for the socio-economic status of the people that live in that area. We then compare that median income of each block group with the distribution of income in the city so each area is assigned to a particular quantile of economic status within each city. Users in our data set are grouped in four equally-size quantiles of income in the city, so each individual is classified as *low income* (bottom 25%), *lower-middle income* (between 25% and 50% median income), *higher-middle income* (between 50% and 75% median income) and *high income* (top 25% in income). Intervals of median income for each economic group and city are shown in Supplementary Figure 2. Individuals' economic status is then the quantile of the census block group where their home cluster is located. It's worth noting here that this method of assigning socioeconomic status partially suffers from an *ecological fallacy*: census block groups have households that vary in income, and the median income of a block group may not be an appropriate descriptor of an individual user – a user in a low-income block group may actually be a high-income individual. However, because our results are robust to changes in quantile definitions (see below and Supplementary Note 1.4), we argue that they are likely robust to this effect as well.

Our income segregation results for places and individuals are robust against other definitions of economic groups. To test it, we have tried 3 or 5 quantiles instead of 4 to classify the different economic groups and generalized the definition of income segregation (described in Supplementary Notes 2.1 and 2.2) to:

$$S_\alpha^{\{n\}} = \frac{n}{2n-2} \sum_{q=1}^{n} \left| \tau_{q\alpha} - \frac{1}{n} \right|,$$

where n is the number of quantiles used. We obtain a (Pearson) correlation of $\rho[S_\alpha^{\{3\}}, S_\alpha^{\{4\}}] = 0.86 \pm 0.01$ between the income segregation for places obtained using 3 and 4 quantiles and $\rho[S_\alpha^{\{4\}}, S_\alpha^{\{5\}}] = 0.89 \pm 0.01$ with 5 quantiles. Similarly, we obtain very high correlations for individual experienced income segregation using different numbers of quantiles. Specifically $\rho[S_i^{\{3\}}, S_i^{\{4\}}] = 0.90 \pm 0.01$ and $\rho[S_i^{\{4\}}, S_i^{\{5\}}] = 0.93 \pm 0.02$.

We have also investigated the potential misclassification of user income groups within a given CBG by using their median household income. In each CBG we actually have a distribution of household income which means that users can belong to different quantiles as the one for the median income [4]. We note first that CBGs are very small and typically include only thousands of individuals and have a small spatial extension. This means that the distribution of income within a given CGB is not very broad and most users (around 60%) belong to the same quantile as the median income. To test the sensitivity of our results to the actual distribution of income within each CBS we have assigned each individual an income drawn from the distribution of income within their home CBG. After that we divided the individuals into four quantiles of income and recompute the place and individual income segregation using these new groups. We run 100 realizations of this stochastic income assignment and compare the original place and individual income segregation with the average of those 100 realizations. We found that place and individual income segregation are correlated with the original ones ($\rho = 0.796 \pm 0.001$ for places and $\rho = 0.839 \pm 0.001$ for individuals).

Finally our results are also robust to other proxies of socio-economical status. For example, if we use quantiles of the ratio of poverty level in each census block instead of the median household income, we get a large correlation of $\rho[S_\alpha^{\{income\}}, S_\alpha^{\{poverty\}}] = 0.72 \pm 0.01$ for the income segregation at each individual place and $\rho[S_i^{\{income\}}, S_i^{\{poverty\}}] = 0.75 \pm 0.01$ for individual income segregation. All these robustness checks show that our particular definition of socio-economical status and groups do not significantly change our results.

1.4 Representativeness of the data

Our location data comes from smartphones in large urban areas and although 83% of the U.S population owns a smartphone in urban areas [5], we might question whether our sample of 3.6 million individuals is representative of the distribution of income in cities, specially for lower-income individuals, or if our dataset allows us to accurately determine how many and which income groups visit a particular place. We have tested both of these questions in different ways.

1.4.1 Population representativeness

Supplementary Figure 3a shows the comparison between the population detected in our data and the 2012-2016 5-year ACS for each of the CBGs in the 11 metropolitan areas. As we can see, the correlation between them is moderately high ($\rho = 0.65$) showing that despite the use of such small census areas, we still get a good representation of the population. Despite that, we address the representativeness of the data using a weighting mechanism (post-stratification) based on the ratio of smartphone users to the true population in the block group. Post-stratification is a well-known sampling tool [6] and is typically used from observational data coming from mobile phone data [7] or social media [8] to study transportation, mobility or income segregation in cities.

Let's denote w_g the ratio of the population of census block g to the population detected in our data. For census block groups with fewer than 7 smartphone residents, the expansion factor w_g is set to 0 to ensure that we do not overweight users that are not representative for a given census block. Then we can weight the time people from census g spends in venue α by

$$\hat{\tau}_{g\alpha} = w_g \tau_{g\alpha}$$

where we are making the assumption that $\tau_{g\alpha}$ is proportional to the number of people visiting the place.

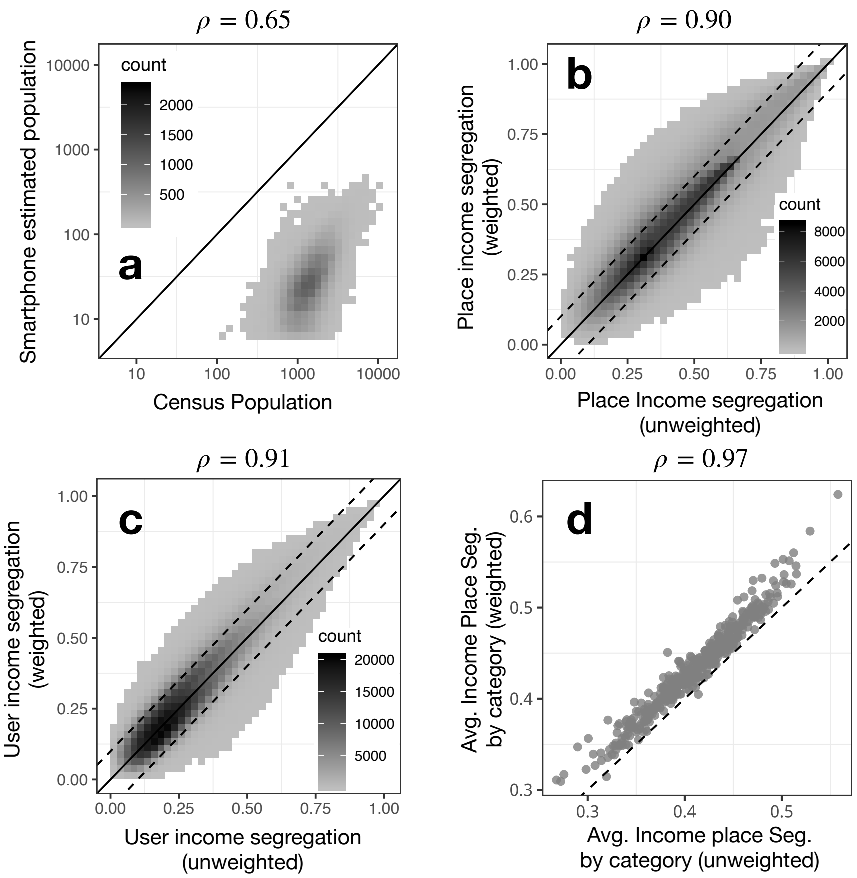

Supplementary Figure 3: Correlation between the smartphone population detected in our data and Census population. a) Heat-map of the census block group population detected in our smartphone data vs. Census 2012-2016 ACS. (b) Heat-map of place income segregation using the weighted and unweighted visits to the place. Dashed line correspond to 10% bounds of difference between them. (c) Heat-map of user experienced income segregation using the weighted and unweighted segregation within venues. (d) Comparison between the average place income segregation by category with and without weighting visits by venue. Source data are provided as a Source Data file.

Then we can recompute the amount of time people of quantile q going to venue α as

$$\hat{\tau}_{q\alpha} = \sum_{g \in \mathcal{G}_q} \hat{\tau}_{g\alpha} = \sum_{g \in \mathcal{G}_q} w_g \tau_{g\alpha}$$

where $\mathcal{G}_q$ is the set of census block groups with median income in quantile q. Supplementary Figure 3b/c show how venue and user income segregation change when $\hat{\tau}_{q\alpha}$ is used instead of the unweighted (raw) data $\tau_{q\alpha}$. As we can see the results are very similar (Pearson's correlation $\rho =\simeq 0.9$) and 88% (users) and 99% (venues) of income segregation values are within 10% difference. This high correlation between unweighted and weighted user/venue income segregation translates only into slight differences in the main results in the paper. For example in Fig. 3d we show the correlation between the average place segregation by category with and without the weighting. As we can see both the values are very similar.

1.4.2 Income representativeness

Since our calculations depend on the income distribution within metropolitan areas, we should make sure that the difference between our sample of users and the real population does not encode a particular bias

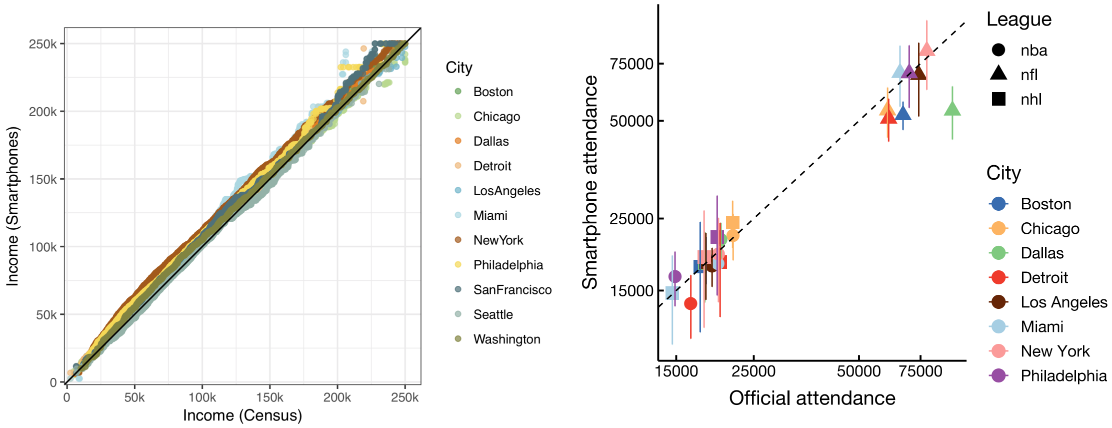

Supplementary Figure 4: Left: Quantile-quantile plot of the distribution of income from the total population (Census) and our sample (Smartphones) for the different cities (CBSAs). Right: Official average attendance to major professional sports games (NFL, NHL and NBA) in 8 cities compared to the detected average attendance during seasons 2016-17. Vertical bars are 95% confidence intervals for the smartphone data over all the games by league and city. Source data are provided as a Source Data file.

towards a particular income group. Supplementary Figure 4 compares the distribution of income in each city extracted from the Census with that of our sample of smartphones. As we can see the relationship is very close, although our sample is a little bit shifted toward higher incomes. In particular we get that the average income of our sample of users is 8.6% higher than the census data, excepting Seattle where our sample has 0.1% lower income.

We have also checked that our results do not depend on whether the quantiles are computed using the actual distribution of income in the city from the census or the detected distribution of income of the users in our dataset. As Supplementary Figure 4 suggests the definition of those quantiles is very similar. For example in Boston we get that the quantiles are $[0, 67k], [67k, 90k], [90k, 114k]$, and $[114k, 250k]$ using our sample of users, while we get $[0, 59k], [59k, 84k], [84k, 108k]$, and $[108k, 250k]$ from the census. In all the cities we get that around 85% of the users belong to the same quantile group in both definitions. Finally we get that our main results in the paper, individual and place income segregation, are correlated for both definitions of quantile groups. In particular we get that $\rho[S_\alpha^{\{\text{dataset}\}}, S_\alpha^{\{\text{census}\}}] = 0.86 \pm 0.01$ for place income segregation and $\rho[S_i^{\{\text{dataset}\}}, S_i^{\{\text{census}\}}] = 0.85 \pm 0.01$ for individual income segregation.

These results shows that our dataset does not have significant biases in income distribution by city and that our results are not affected by the definition of income groups.

1.4.3 Comparing official attendance to professional sports games

We have also checked the representativeness of the data at the level of individual venues. To do this, we compute the attendance to games of the major professional sports leagues in 8 cities. In particular, we calculate how many different individuals have a stay within a stadium's perimeter. We do this for a variety of games in the National Football League (NFL), National Basketball Association (NBA), and National Hockey League (NHL). Attendance for each particular game is calculated using stays within the stadium happening 3 hours before the starting time up to 3 hours after the game is finished. Since penetration of our smartphone data varies across cities, we have used a normalization factor $\rho_c = S_c/N_c$, the ratio of our smartphone population S_c to the total population N_c in the area c. Thus, if $S_{\mathrm{i,c}}$ is the average number of users detected in the stadium for sport i's games, we assume that the observed attendance goes like $\mathrm{Att}_{\mathrm{i,c}}^{\mathrm{obs}} = aS_{\mathrm{i,c}}/\rho_c$, where a is a constant for all cities and all sports to account, among other things, for the daily (lower) penetration of individuals detected compared to the whole 6 months period. In our case we have taken $a = 2.6$. Supplementary Figure 4 shows that the values of attendance obtained from our

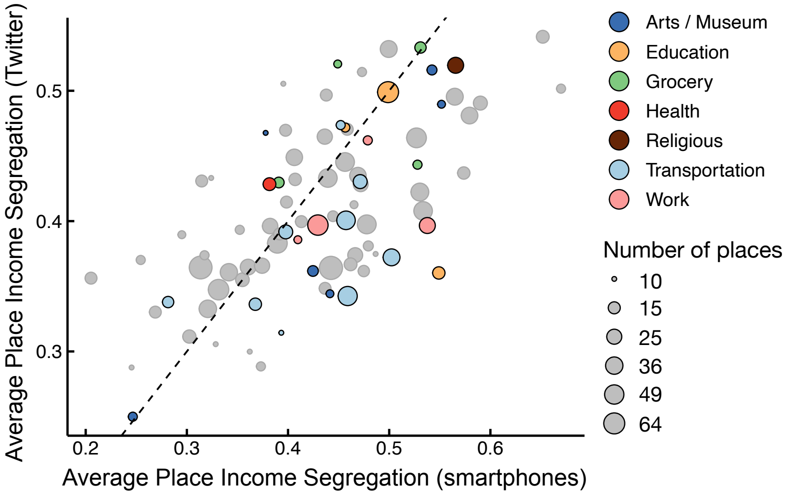

Supplementary Figure 5: Comparison between the average income segregation by place category using our data (smartphones) and Twitter data. Each point correspond to a different category, colors to different type of categories and size to the number of places identified in the Boston metro area. Correlation between the different datasets is $\rho = 0.70 \pm 0.11$. Source data are provided as a Source Data file.

data coincide with the official figures. The only exception is the average attendance to the Dallas Cowboys games, possibly because a large fraction of people attending the game might come from areas outside the Dallas CSBA considered. This remarkable results shows that our data describes quite well even visits to a particular venue.

1.4.4 Other datasets

Although our dataset is unique in terms of number of users, time and space resolution of human mobility, we have compared our results with other geolocalized datasets to check the validity of our results. In particular, we have use a dataset of 3 million geolocalized tweets from 109k unique users in the Boston metro area, collected during the first 6 months of 2014, when geolocalized tweets where still numerous. Contrary to our dataset, (i) geolocalized tweets are more sparse, (ii) do not give information about time spent in a place, and (iii) were collected in a different time window. Using the same methodology, we have computed place segregation for only the 2605 places which were visited by at least 15 twitter users. We have assume that each tweet correspond to a stay with a duration of one time unit. Despite the problems mentioned before, correlation between place segregation using our data and Twitter data is moderate $\rho[S_\alpha^{\text{Twitter}}, S_\alpha^{\text{smartphones}}] = 0.51 \pm 0.12$ maybe due to the large noise in the Twitter data because of its sparsity. However, some of the main aggregated results (like those in Figure 2 of the main paper) are still valid in the Twitter data. Supplementary Figure 5 shows that our result about the dependence between place category and income segregation still is valid when we use Twitter data. This finding shows that our results are not an artifact of specific biases of our dataset. Given the sparsity of the Twitter data we could not get reasonable and statistical robust results for individual income segregation.

2 Measuring Place and Individual Income segregation

2.1 Place income segregation

To measure the income segregation of each place α in each city, we compute the proportion of total time spent at that place α by each income quartile q, $\tau_{q\alpha}$. To control for variation in incomes between cities,

we define separate quartile gradations for each city in our dataset. We define full integration of a place as $\tau_{q\alpha} = 1/4$ for each q, that is, the total time spent at venue α is split evenly across our four income quartiles. We then define the income segregation for each place α, S_α, as any deviation from our idealized measure of integration:

$$S_\alpha = \frac{2}{3} \sum_q \left| \tau_{q\alpha} - \frac{1}{4} \right|. \tag{1}$$

The measure S_α is bounded between 0 and 1. A place with $S_\alpha = 0$ means that a venue α is visited equally by all income quartiles in the city, with no deviation from our idealized integration measure of $\tau_{q\alpha} = 1/4$. By contrast, a venue with $S_\alpha = 1$ is one that is visited exclusively by a single income group. Therefore, a higher S_α measure indicates that a place is visited more exclusively by a single income group, hence a higher level of income segregation. Our metric of income segregation is very similar to other typical segregation measures like the entropy or interaction coefficient within a place [9], and our results are robust to changes in how income segregation is defined (see Supplementary Note 3). Note that because our income groups are defined by population quartiles, S_α is defined relative to the actual household income distribution in each CBSA.

2.2 Individual experienced income segregation

If $\tau_{i\alpha}$ is the proportion of time individual i has spent at place α, then we can define a individual's relative exposure to income quartile q, τ_{iq}, as a sum over all places α visited by individual i: $\tau_{iq} = \sum_\alpha \tau_{i\alpha}\tau_{q\alpha}$, where $\tau_{q\alpha}$ represents the proportion of time at place α spent by income group q. This effectively represents the probability that an individual is exposed to income group q in their daily behavior. Using this measure, we can then define individual experienced income segregation, S_i, as a simple rewriting of Eq. (1): $S_i = \frac{2}{3}\sum_q \left|\tau_{iq} - \frac{1}{4}\right|$. Our metric for individual income segregation can be thought of as an extension of the traditional metric of isolation or interaction for groups to the level of individuals based on daily encounters among them [9].

Note that while the mobility data set we use is large, co-location events between individuals are still quite sparse. Because of this sparsity, and to protect individual privacy in our analysis, we have adopted this probabilistic approach to measuring encounters. Specifically, we have made the assumption that the instantaneous distribution of income groups q in place α can be approximated by its temporal average $\tau_{q\alpha}(t) \simeq \tau_{q\alpha}$ so if a user visits place α a number of times it would be exposed to the different groups with probability $\tau_{q\alpha}$. However if users from a given group visit the place at particular times or if the instantaneous distribution $\tau_{q\alpha}(t)$ fluctuates wildly, the actual exposure of user i to the different groups in place α can be very different from $\tau_{q\alpha}$.

We test this hypothesis by using the actual co-location events of the different users in our dataset. Each time a user visits place α we detect the number of users co-located with i during their visit. By aggregating over all the visits of user i to place α we calculate $\hat{\tau}_{iq\alpha}$, the fraction of users from group q co-located with user i at place α. In our approximation we assume $\hat{\tau}_{iq\alpha} \simeq \tau_{q\alpha}$ and as we can see in Supplementary Figure 6 this is a very good approximation. In fact the correlation between both quantities is $\rho = 0.701 \pm 0.004$ and 80% of the values are within 15% absolute difference. This result suggest that $\tau_{q\alpha}$ is a good approximation of the distribution of income groups a user encounters in place α and thus, given the sparse nature of co-location events, we have decided to use it to compute individual income segregation.

Individual experienced income segregation depends on the amount of time an individual spends in places. Our results and the model in Supplementary Note 4 (see Supplementary Figure 10) show that individuals visit many places for short time periods, while spending most of the time in a very small set of places which might be less consequential for the individual. Which part of the individual income segregation comes from the places they go regularly or from fleeting encounters in least visited places? To test this, we have calculated the individual segregation $S_i^{(N)}$ using only the top N most visited places by each user (in duration). As we can see in Supplementary Figure 7A the average individual income segregation in our cities obtained by measuring it only at the top N places is very similar to the total result, even for small values like $N = 5$. The reason for that is that individuals spend most of their time in a small number

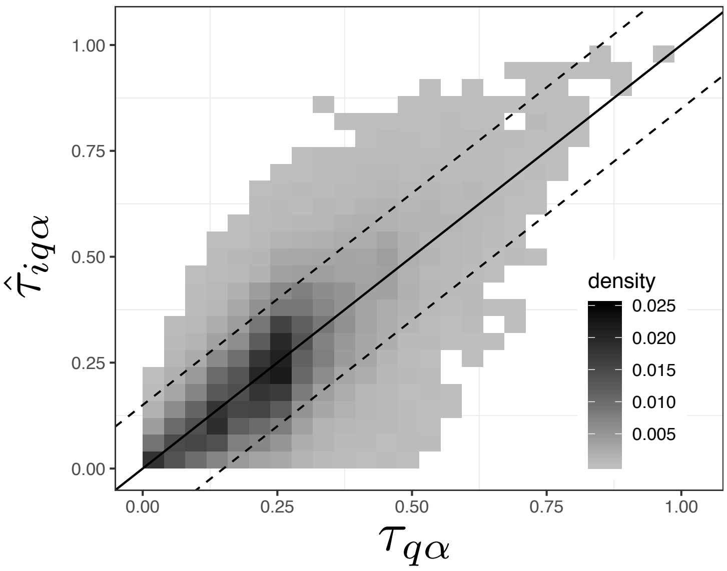

Supplementary Figure 6: Heat-map of the values of the actual distribution of income groups q co-located with user i in place α and our probabilistic approximation using the temporal average $\tau_{q\alpha}$. To get good estimates of $\hat{\tau}_{iq\alpha}$ we only show those values for places α that were visited more than 15 times by user i. Dashed lines correspond to 15% absolute difference between the actual distribution and our approximation. 82% of the data is contained between dashed lines. Only data for the Chicago area is shown. Source data are provided as a Source Data file.

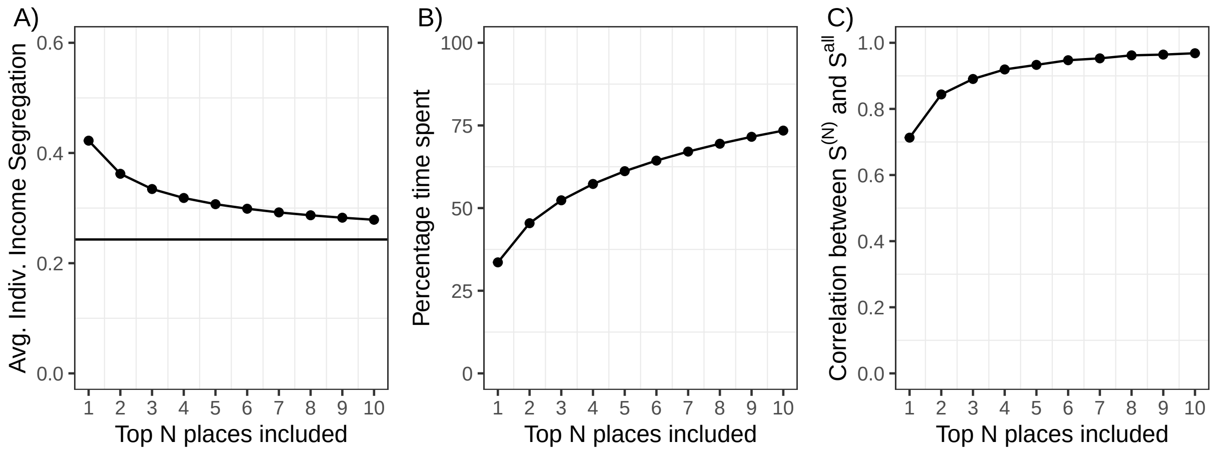

Supplementary Figure 7: A) Average individual income segregation in our cities computed using only the top N places visited by an individual. The horizontal line is the value when all places are considered. B) Average percentage of stays duration by individual when only the top N places are included. C) Correlation between individual income segregation obtain with only N top places and all places. Source data are provided as a Source Data file.

of places. We obtained a correlation of $\rho[S_i, S_i^{(N)}] = 0.969 \pm 0.0002$ when $N = 10$ which agree with our assumption that the user income segregation is effectively calculated on the most visited and thus more consequential places for each user. Supplementary Figure 7 shows the correlation between them.

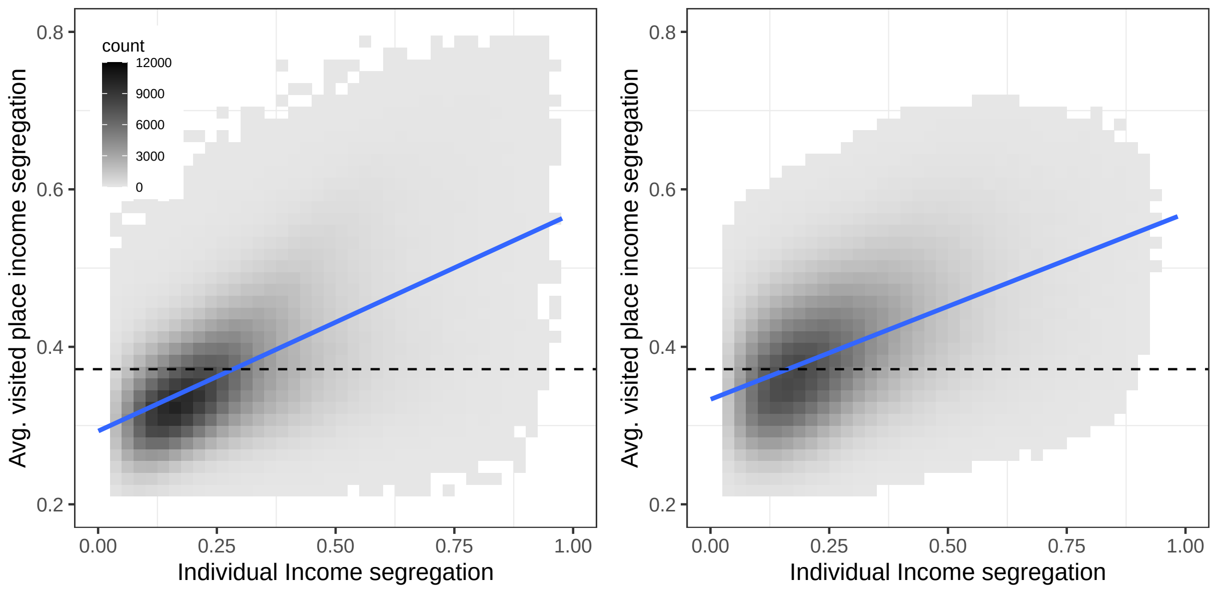

Supplementary Figure 8: Left: Comparison between individual experienced income segregation and the average income segregation of the places visited by the individual in our dataset. B) Right: Comparison between individual segregation and the average segregation of the places visited by the individual in our social-EPR model. Dashed line is the average income segregation of places in our cities. Note that highly integrated (low income segregated) individuals) tend to visit less (than city's average) income segregated places while highly segregated individuals tend to visit more income segregated places. Source data are provided as a Source Data file.

2.3 Relationship between individual and place income segregation

We note that individual income segregation experience S_i is not a mere weighted sum (by duration) of the income segregation of places visited by individual i. In fact, S_i is a convoluted version of the S_α for all α visited by i that represents the overall likelihood that a user encounters people of different backgrounds. Although we expect that highly income segregated individuals visit mostly income segregated places, in principle it is possible that a person with low overall income segregation flits between income segregated places dominated by different socioeconomic groups.

We have tested this by computing the correlation between S_i and $\langle S_\alpha^i \rangle_i$, the average income segregation of the places visited by user i. We see a strong correlation between them $\rho[S_i, \langle S_\alpha^i \rangle_i] = 0.579 \pm 0.001$ (see Supplementary Figure 8), meaning that users that are highly integrated tend to visit highly integrated (at least more than the city's average) places. Thus, the expected behavior for highly income segregated individuals is also seen for low income segregated ones: individuals visit places as segregated as their overall individual segregation.

This result can be explained within our simple social-EPR model (see Supplementary Note 4) by the null hypothesis that individuals visit randomly places in their group majority or not among the ones available to them. For individuals with high S_i, places visited are mostly segregated (towards their group) and thus the average income segregation is high. For small S_i, individuals visit mostly places with no majority group and thus the average income segregation is low. In our social-EPR model we get that $\rho[S_i, \langle S_\alpha^i \rangle_i] = 0.496 \pm 0.001$ (see Supplementary Figure 8), confirming that the relation between S_i and the average income segregation can be explained by our simple model.

3 Other measures of income segregation

Although there are many different dimensions to measure economic inequality in urban areas [9], our metric of inequality at places and of users a simple extension of the idea of the *unevenness of exposure* between different groups in a place or of a particular individual to the groups in the city. Other traditional metrics of unevenness or exposure are well suited when the full distribution of income is known (Gini coefficient, Theil index) or when only interaction between two groups (typically majority/minority, isolation and interaction indexes) is considered. Moreover the calculation of some of these metrics to a particular place or a sole individual require complicated extensions to account for each city income distributions or absence of groups in particular places.

To this end we have adopted a metric [see Eq. (1)] that measure the relative unevenness of the distribution of quantile groups present in a place normalized to distribution of income in the city. Note that unevenness is measured using the L1-norm (absolute distance) instead of other options like the Euclidean distance $S_\alpha^{(eucl)} \sim \sum_\alpha (\tau_{q\alpha} - 1/4)^2$. In our data we found that $\rho[S_\alpha^{(eucl)}, S_\alpha] = 0.9677 \pm 0.0004$ so this choice does not change our results.

Another possible metric to measure this unevenness of the distribution of groups is the *entropy* [9]. In the case of places it can be calculated like

$$H_\alpha = \frac{1}{\log 4} \sum_{q=1}^{4} \tau_{q\alpha} \log \tau_{q\alpha}$$

Note that when all groups are equally present at place α then $\tau_{q\alpha} = 1/4$ and thus $H_\alpha = 1$. However when only one group visit α then $H_\alpha = 0$. Supplementary Figure 9a shows the high correlation between our metric of income inequality and the traditional metric of entropy (by place and by average category). In fact we get that $\rho[S_\alpha, H_\alpha] = -0.972 \pm 0.001$ for the 1.01 million places in our dataset. Despite this, we have used our metric instead of the entropy because of its simplicity and its larger variability in the $[0, 1]$ domain which helps in the visualizations and in the modeling (see Supplementary Figure 9b).

A third, traditional metric of unevenness of exposure is that of interaction which measures the probability high income groups are exposed to low income groups. In our case it could be defined as:

$$I_\alpha = 4 * (\tau_{1\alpha} + \tau_{2\alpha}) \times (\tau_{3\alpha} + \tau_{4\alpha})$$

where we have define the two groups as the union of the two high and low income quantiles. As with the entropy we find a large correlation of our results with the place interaction ($\rho[S_\alpha, I_\alpha] = -0.805 \pm 0.001$) and average interaction by category (see Supplementary Figure 9).

As a final choice, we have also considered Pythagorean distance in a four-dimensional space as a measure of income segregation. We found that the Pythagorean distance is correlated with our measure of segregation ($\rho = 0.97 \pm 0.01$).

Similar results are found for individual segregation when entropy, interaction, or Pythagorean distance are used instead of our definition of segregation. This demonstrate that our results are robust against the specific type of distance to calculate income segregation of places or individuals.

4 The social exploration and preferential return (social-EPR) model

4.1 Definition of the model

In the EPR model each time an individual visits a place, it is a new one with probability $P_{\rm new}$ or the individual returns to a previous visited place with probability $1 - P_{\rm new}$. According to the EPR model, $P_{\rm new} = \rho S_n^{-\gamma}$, where S_n is the number of unique places the individual has visited up until visit n. For places that have already been visited, the probability that an individual i visits a place α, Π_α, is proportional to the amount of time that individual has spent there in the past, $\tau_{i\alpha}$. We validate these hypotheses and find that, when we fit the model's parameters to our data, we obtain $\gamma \simeq 0.23 \pm 0.02$ which is similar to what has been reported

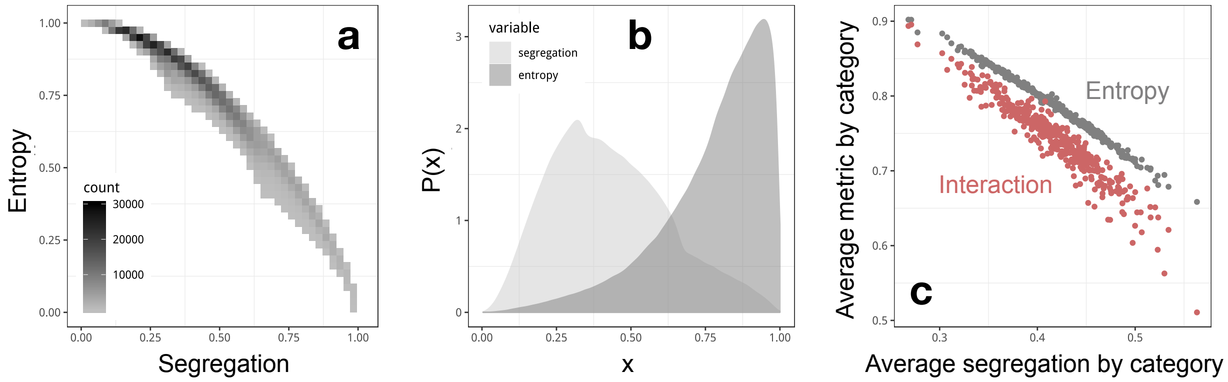

Supplementary Figure 9: Comparison of our income segregation metric with other metrics: a) Correlation between our metric of income segregation with the traditional entropy metric for each of the 1.01 million places in our dataset. b) Density of the values of our metric of segregation and entropy for the different places. c) Comparison between the average segregation by category of places (see Figure 2 in the main text) and the average entropy and interaction. Source data are provided as a Source Data file.

in a few other studies of urban mobility[10] (see Supplementary Note 4). The information contained in ρ can be equivalently captured by an individual i's *place exploration*, which we define as $\sigma_p = S_T/N$, where S_T is the number of unique places i has visited and N is the total number of stays for i (see Supplementary Note 4).

4.2 Testing the hypothesis of the model

Using the sequence of visits for each user we are able to check the hypothesis of the model. In Supplementary Figure 10a we can see that, as expected, $P_{\text{new}}(S) \simeq \rho S^{-\gamma}$ where $\gamma = 0.22 \pm 0.01$ and as expected [see equation (2)] the value of ρ (vertical shift in Supplementary Figure 10) increases with total number of places explored S_T. Note that the values obtained (in particular γ) are consistent with the finding in [10] that $\gamma = 0.21 \pm 0.02$ obtained with a completely different dataset of human mobility in urban areas. This finding supports the representativennes of our data as well. We also found that the preferential return mechanism $\Pi_\alpha \propto \tau_{i\alpha}$ is accurate (see Supplementary Figure 10b), although there is a little bit of deviation from the straight line.

The EPR model predicts that individuals visit specific places with a total time t_α or frequency τ_α that follows Zipf's law, i.e., $P(\tau_{i\alpha}) \sim 1/\tau_{i\alpha}^{\eta}$ with $\eta = \frac{2+\gamma}{1+\gamma}$ [10], a result which is confirmed by our data (see Supplementary Figure 10c and Figure 3 in the main text) and reflects the accuracy of the model to describe the mobility of individual users in the city. Note that since $\frac{2+\gamma}{1+\gamma} \simeq 1.82$ in our case, the distribution of $\tau_{i\alpha}$ is heavy tailed, i.e., people spend most of their time in a very small set of places. In our data, we find that on average, 80% of people's time is spent in only 9.84 ± 0.04 places. This result partially explains why the average number of unique people an individual encounters in the city is very small. Across all 11 cities, we find that on average people only encounter around 4000 other unique people in 6 months.

4.3 Measuring the parameters of the social-EPR model

The social-EPR model for each individual is then defined by two parameters:

- ρ, which controls P_{new} and in turn the total number of different places visited by the user S_T, i.e. the amount of exploration behavior of the user. In particular we have that [10]:

$$S_T = [1 + \rho(1+\gamma)N]^{1/(1+\gamma)} \tag{2}$$

We encode this information into the parameter $\sigma_p = S_T/N$, which measures the fraction of total visits to new places.

- σ_s, which measures the fraction of places visited by the user where their income group is the minority.

4.4 Simulating the social-EPR model

To test the validity of our model to explain income segregation experienced by users, we have simulated it for each of 1.03 million users in our dataset that have more than $N > 50$ visits. For each user within a given income group (quantile) we extract the actual values of N (the number of visits), σ_p the fraction of visits to new places and σ_s the fraction of unique places visited where the user's income group is the minority. With N and σ_p and inverting equation (2) we get the value of ρ for each user.

Then the simulation for each visit $n = 1, \ldots, N$ creates a sequence of places according to the following algorithm:

1. At $n = 1$ we place the user in their most visited place obtained from the data. We assume that the user spent initially two units of time there.

2. For $n > 1$ with probability

   - $P_{\text{new}} = \rho/S_n^\gamma$ (where S_n is the number of different places visited up to visit n) a new place to visit is chosen. The new place is selected:
     - with probability σ_s from those in the city in which the user's income group is not in the majority
     - with probability $(1 - \sigma_s)$ the new place is selected from those in the city in which the user's income group is in the majority.

     After selecting the new place we set the amount of time spent there to one time unit.
   - (1-P_{new}) the user returns to a previously visited place with probability $\Pi_\alpha \propto \tau_{i\alpha}$ where $\tau_{i\alpha}$ is the amount of time already spent in place α. We increment the time spent of the chosen place by a unit of time.

Note that for simplicity our simulations sample the new places visited by each user from the pool of all places in the city. Much more refined models can be built that account for individual mobility patterns [7] or gravity models [11] to select the new places.

With the given sequence of places visited by user i we compute its individual income segregation using the simulated $\tau_{i\alpha}$ (fraction of time spend by users i in place α) and the real $\tau_{q\alpha}$ (fraction of time users of quantile q spend in place α). Note that we could have used our simulations also to compute $\tau_{q\alpha}$ by place. However, this would lead to large statistical fluctuations of place income segregation patterns for each stochastic realization of our simulations. Thus we have assumed that $\tau_{q\alpha}$ is fixed and given by the real data.

Despite its simplicity, our model is able to account for 60% of the variability of individual income segregation. Indeed, the correlation between the real individual income segregation and the simulated one is $\rho[S_i, \hat{S}_i] = 0.777 \pm 0.001$.

In our model, users visits new places in a similar fashion as the Schelling model for spatial income segregation: with probability $1 - \sigma_s$ they go to a place in the city in which their user income group is in the majority. This is defined as at least 50% of the people visiting the place belongs to the same income group. To test how our results depend on that threshold we have simulated the social-EPR model for different thresholds. As we can see in Supplementary Figure 11, the correlation between the results of individual income segregation for the social-EPR model and the real data is optimal around that 50% threshold. For simplicity we have used 50% instead of the actual value for which the correlation is maximized.

Our model also makes the assumption that the income segregation pattern of places is chosen with probability σ_s, independently of the time spent there. Our data corroborate this assumption: correlation

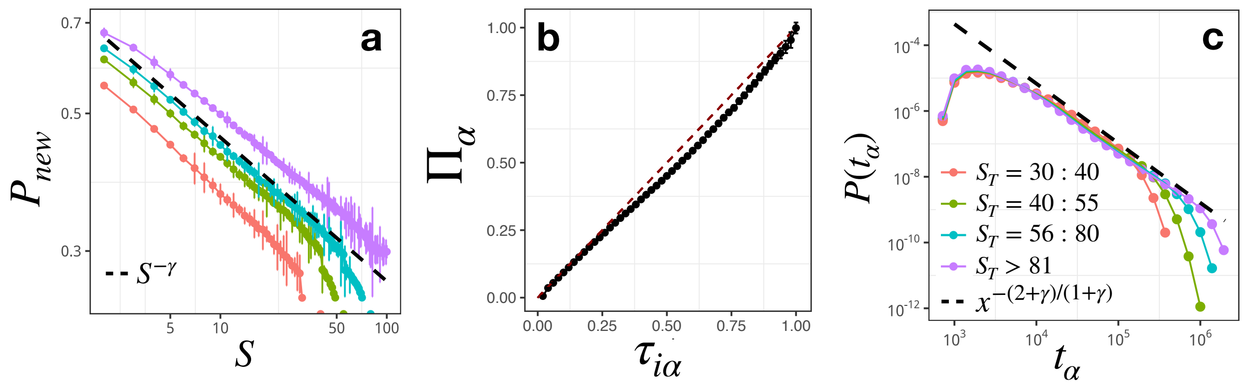

Supplementary Figure 10: Testing the hypothesis and results of the EPR model. (a) Probability of visiting a new places as a function of the number of already visited places for different groups of users (see panel c for colors). Each group correspond to a different total number of visited places and the dashed line is a power-law fit indicating that $P_{\text{new}} \sim S^{-\gamma}$ with $\gamma = 0.23 \pm 0.01$. Vertical lines correspond to the 95% confidence intervals for the probability for each group and given S. (b) The probability Π to return to a previously visited location α as a function of the previous frequency of visitation $\tau_{i\alpha}$. Dashed line correspond to the line $\Pi \sim \tau_{i\alpha}$ and vertical bars are 95% confidence intervals for the probability. (c) Distribution of total time spent in each place t_α for the same groups as in the a) panel. The dashed line is the prediction of the social-EPR model $P(t_{i\alpha}) = t_{i\alpha}^{-(2+\gamma)/(1+\gamma)}$ with $\gamma = 0.22$ chosen from the fit in panel a). Source data are provided as a Source Data file.

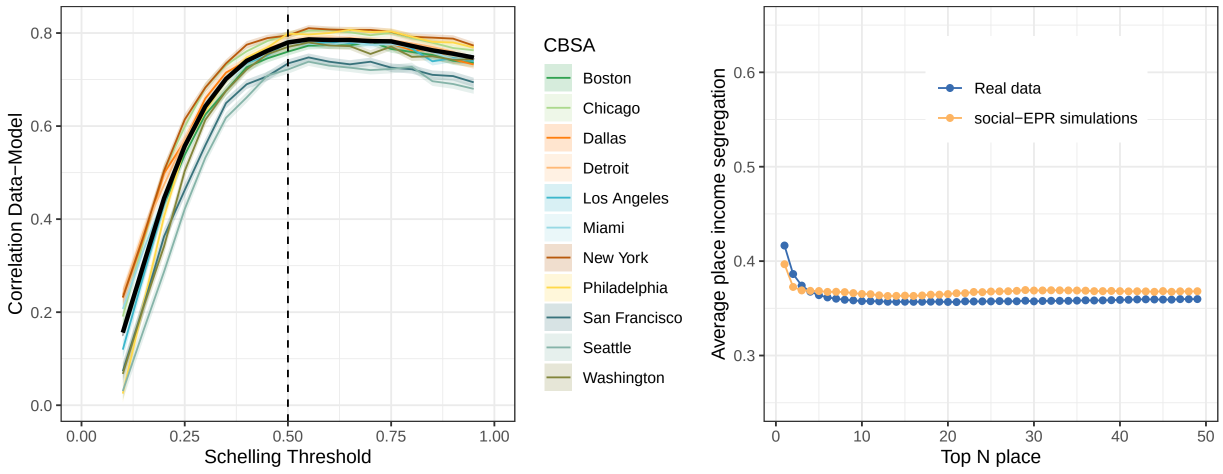

Supplementary Figure 11: (Left) Dependence on the Schelling threshold: correlation between the real individual segregation and the one obtained in the simulation of the social-EPR model for different values of the Schelling parameter and different cities (colors). Vertical line shows 50%, the value used in our final model and black line is the correlation for the full dataset. Shaded ribbons are 95% confidence intervals for the correlations. (Right) Average income segregation for the top N places in the real data and the effective one in our social-EPR simulations. Source data are provided as a Source Data file.

between the segregation of places and the time spend there is small ($\rho[S_\alpha, \tau_{i\alpha}] = 0.049 \pm 0.001$), see Supplementary Figure 11 where we can see that S_α is almost the same for each top place. However, we observed in general that top places are slightly more segregated than the rest. By choosing the most visited place as the initial condition in our social-EPR model we are effectively reproducing this effect in our simulations, see Supplementary Figure 11.

5 Model for Place Income Segregation

Place income segregation is modeled according to simple linear regression model for all categories

$$S_\alpha \quad \sim \quad \mathrm{Cat}_\alpha + \mathrm{Rating}_\alpha + \mathrm{Price}_\alpha + \mathrm{Catchment}_\alpha + \mathrm{Neigh}_\alpha + \mathrm{Area\_Income}_\alpha + \mathrm{PUMA}_\alpha \tag{3}$$

where:

- Cat_α is the category of the place (Grocery Store, Convention Center, Office, Chinese Restaurant, etc)
- Rating_α is the rating (from 0 to 10) of place α in the Foursquare platform.
- Price_α is the price tier (from $ to $$$$).
- $\mathrm{Catchment}_\alpha$ is the average distance from home the individuals travel to get to place α. Due to its large heterogeneity we have use the logarithm of this variable.
- Neigh_α is the number of places close to α within a 100 meters radius.
- $\mathrm{Area\_Income}_\alpha$ is the median household income of the area (Census Block Group) where place α is located.
- PUMA_α is a fixed effect controlling for the PUMA area in which place α is located.

Variables Cat_α, Rating_α and Price_α and obtained using the Foursquare API. Since Rating_α and Price_α are only available for restaurants, we have two different models for all places (excluding rating and price) and only for restaurants. Due to limitations in the Foursquare API we could only get ratings and prices for the venues in Chicago.

To avoid over-fitting for small areas or rare categories, we have only considered venues that belong to a category with more than 10 different places or are in a PUMA area in which we can find at least 10 different places. Results for both models (including or not rating and price) are presented in Supplementary Table 3.

6 Model for Individual Experienced Income Segregation

To understand the effect of each group of variables in explaining individual income segregation S_i or the social ($\sigma_{s,i}$) and place ($\sigma_{p,i}$) exploration, we have build different simple linear regression models of the form

$$S_i, \sigma_i \sim \{R_i\} + \{P_i\} + \{M_i\}$$

where

- $\{R_i\}$ is the set of all the residential variables from the census that describe the demographic, transportation, education, race, employment, income, etc. characteristics of the Census Block Group where i lives. A complete list of them can be found in Supplementary Table 1 grouped by the different ACS tables. Within each group (ACS table) we have discarded some of the variables (for example *Bike* in *Means of Transportation*) due to their large correlation with other variables. There are 29 variables in this group.
- $\{P_i\}$ are variables related to the fraction of time user i spends in each category. Although there are 715 different venue categories in our dataset, we have only considered in our models the 448 categories which have at least 100 venues. Most important categories can be found in Supplementary Table 2. To account only for the most visited places we have taken a binary approach in which the $\{P_i\}$ variables are one (1) if the fraction of time spent in that category is bigger than 0.3% of the time or zero otherwise. This threshold method (and other variants) are traditionally used in this kind of sparse and highly-skewed human data [12] to minimize the effect of the noisy nature of heavy tailed distribution of human activity. The value 0.3% corresponds to the average of fraction of time spent by our individuals in a particular category. We have also checked that our results do not depend strongly on this value. There are 448 variables in this group.

- $\{M_i\}$ are the two geographical mobility variables considered: (i) radius of gyration of the venues visited and (ii) average distance travelled to those venues from each individual's home. There are only 2 variables in this group.

Similar to Supplementary Note 4, in our models for individual income segregation we have only considered the 1.03 million users with more than 50 stays.

To evaluate the relative importance of each group of variables we have used the simple approach of Lindeman, Merenda and Gold (LMG) [13] to each group: if A, B and C are groups of variables and

$$R^2(B|A) = R^2(B \cup A) - R^2(A)$$

is the additional R^2 when the B variables are added to the model with only A variables, the importance of group of variables in group A is the average of additional R^2 obtained by adding the A in any sequential order to introduce each group of variables. In our case we have 3 groups with 6 different sequential permutations which yields to:

$$\mathrm{LMG}(A) = \frac{1}{6}\left[2R^2(A) + R^2(A|B) + R^2(A|C) + 2R^2(A|BC)\right]$$

Results for each model R^2 for each group of variables are shown in Supplementary Table 4 (S_i), Supplementary Table 5 ($\sigma_{s,i}$), and Supplementary Table 6 ($\sigma_{p,i}$).

6.1 Independence of residential and places variables

In principle, residential demographics could have a large impact in individual's lifestyles and thus on the type of specific places visited as well as its frequency. Thus, it is possible that in our models some of the $\{P_i\}$ can be explained by the residential variables $\{R_i\}$. However, as we found σ_s is explained mostly by residential variables and σ_p by place (mobility behavioral) variables (see Figure 4 in the main text). This results show implicitly that both set of variables are largely independent. To make this statement more explicitly we have done different tests:

- We have tried to model each place variable as a function of the residential variables, i.e. P_i $\{R_i\}$ (excluding PUMA area). Those models have small explanatory power (average $R^2 = 0.0068$) which shows the small correlation between time spent in places and residential variables.

- We have also computed the Variance Inflation Factor (VIF) for the residential and place variables in our model for individual income segregation to test for potential multicollinearity. In our models the VIFs range from 1 to 4 indicating that there is no significant issue of multicollinearity.

- Finally we have used simple regularization techniques like LASSO to check what variables are more important in our models. If $\{P_i\}$ and $\{R_i\}$ are related and redundant, then LASSO regularization should have a dramatic effect on the number of variables selected from each group. However, our LASSO model for individual income segregation S_i drops only 19 variables associated with places (out of the 448) and only 1 variable (Retail, in Industry for Employed People) from the residential demographics (out of 29). This results means that the rest of variables contains relevant and not redundant information in our models.

7 Venue categories

We have categorized the venues visited by individuals using the Foursquare classification. We have also grouped manually the venues in our own Taxonomy of 13 groups, Art / Museum, City / Outdoors, Coffee / Tea, College, Entertainment, Food, Grocery, Health, Religious, Education, Service, Shopping, Sports, Transportation, Work (see Supplementary Table 2). The number of stays and time spend in different categories is very different across categories and groups (see Supplementary Figure 12). We found that Work,

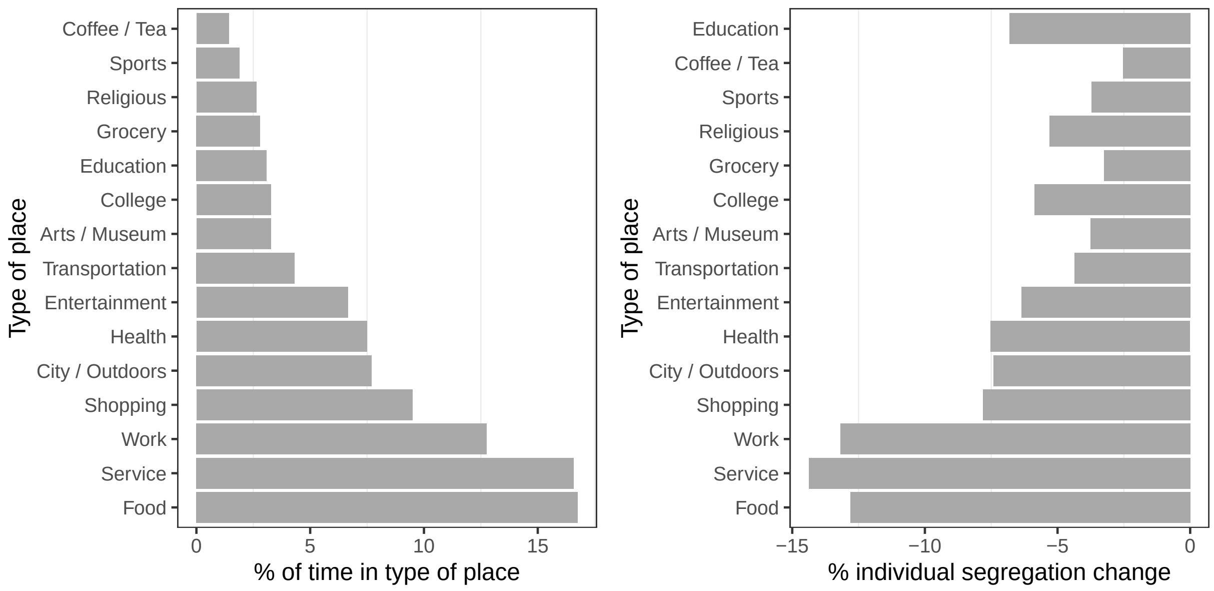

Supplementary Figure 12: (Left) Average fraction of time spend in different groups of venues by users in our database. (Right) Percentage of change on individual experienced income segregation by selecting random venues in a particular type of place. Source data are provided as a Source Data file.

Food, and Service are the groups more frequently visited and where users spend almost half of their time ($\sim 46\%$). The less visited groups are Sports, Religious, Grocery and Education places.

Since users spend more time in certain type of places, user's income segregation experience depends on them. But, how much does individual segregation depend on the specific choice in one of those types? To answer this question we have compared individual segregation with a null model in which individuals visit venues in a particular type randomly across the city. This way we destroy the information about the particular choice made by users in that type, but still retain the percentage of time in each type. This sensibility test shows (see Supplementary Figure 12) that the change in individual income segregation is more or less proportionally to the amount of time spent in each type of place. Hence, certain types of places are more important for individual income segregation because of the amount of time spent there, but not because of individuals' particular choice of venues in those types of places.

8 Software used

Analysis was conducted in R [14] using the following packages:

- Package `data.table` [15] for the load and transformation of the data tables.
- The `h2o` [16] library was used to train the models for place and individual income segregation described in Supplementary Note 5 and Supplementary Note 6.
- Packages `ggplot2` [17] and `leaflet` [18] were used in the visualizations.
- Access to the Census API was done using the `tidycensus` [19] package. Boundaries of the Census Block Groups were obtained from the Census API using the `tigris` [20] package.
- Regression tables were prepared using the `stargazer` package [21].

Supplementary Table 1: Summary statistics of the 29 Census variables used to describe the 55474 Census Block Groups in our 11 Metro areas. Variables were obtained from the different tables of the five-year American Community Survey (ACS) ending in 2016. Each variable represent the fraction of each group within the census block group, excepting the median household income. We have the PUMA area geocode as a fixed effect to account for variation at large scales. Source data are provided as a Source Data file.

ACS Table	Variable (fraction of the total)	Mean	Standard dev.	Min.	Max.
Educational attainment	Below 9th grade	0.051	0.074	0.000	0.671
	From 9th to 12th grade	0.310	0.160	0.000	0.911
	Bachelor degree or more	0.364	0.229	0.000	1.000
Means of transportation to work	Car	0.752	0.235	0.000	1.000
	Public Transportation	0.145	0.194	0.000	1.000
	Walked	0.036	0.072	0.000	1.000
Industry for the employed people	Agriculture	0.005	0.018	0.000	0.745
	Construction	0.057	0.058	0.000	1.000
	Manufacturing	0.087	0.074	0.000	0.733
	Wholesale	0.028	0.034	0.000	0.615
	Retail	0.107	0.067	0.000	0.772
	Transportation	0.054	0.052	0.000	1.000
	Information	0.028	0.036	0.000	0.477
	Finance	0.076	0.062	0.000	0.806
	Professional	0.137	0.084	0.000	1.000
	Educational	0.232	0.102	0.000	1.000
	Arts	0.094	0.072	0.000	1.000
	Public	0.043	0.050	0.000	0.704
Age group	Below 18 yo	0.218	0.086	0.000	0.695
	Between 19 and 25 yo	0.090	0.066	0.000	0.974
	Between 26 and 65 yo	0.549	0.092	0.000	1.000
Employment Status	Total civilian in labor force	0.658	0.112	0.009	1.000
	Employed civilian in labor force	0.607	0.119	0.006	1.000
Race	White	0.634	0.290	0.000	1.000
	Black or African American	0.160	0.255	0.000	1.000
	Native	0.004	0.013	0.000	0.436
	Asian	0.091	0.135	0.000	0.981
Poverty & Income	Median household income	76056.119	41679.485	2499.000	250001.000
	Poverty ratio	0.138	0.138	0.000	1.000
PUMA Area	Fixed effect (GEOID)	-	-	-	-

Supplementary Table 2: Summary statistics of the venues categories used in our models grouped by types of places. Only categories with more than 200 venues are shown. For privacy reasons some categories have been masked. Source data are provided as a Source Data file.

Taxonomy	Category	Number of venues	Avg. Income Segregation	Average Distance	Average # of users	Average number of visits
Arts / Museum	Exhibit	234	0.322	21.801	112.047	186.073
Arts / Museum	Jazz Club	236	0.424	14.069	75.564	192.161
Arts / Museum	Indie	252	0.445	14.273	55.250	144.821
Arts / Museum	Zoo Exhibit	265	0.373	22.800	95.370	148.060
Arts / Museum	Science Museum	280	0.308	23.771	118.171	185.607
Arts / Museum	Art Museum	344	0.372	18.378	101.189	211.387
Arts / Museum	Public Art	440	0.414	17.142	77.539	179.055
Arts / Museum	Arts & Entertainment	444	0.435	16.010	69.405	182.097
Arts / Museum	Photography Lab	503	0.466	13.621	99.308	213.994
Arts / Museum	Museum	546	0.394	17.981	86.136	190.394
Arts / Museum	Rock Club	593	0.436	14.814	68.396	166.526
Arts / Museum	Comedy Club	611	0.424	15.093	73.951	174.607
Arts / Museum	History Museum	621	0.404	18.249	72.142	161.140
Arts / Museum	Arts	660	0.438	13.373	56.191	183.262
Arts / Museum	Concert Hall	841	0.406	17.148	96.473	246.304
Arts / Museum	Performing Arts	1287	0.423	15.428	72.271	193.071
Arts / Museum	Movie Theater	1297	0.355	12.976	211.658	398.085
Arts / Museum	Music Venue	2104	0.448	16.050	72.871	185.952
Arts / Museum	Arts & Crafts	2170	0.403	12.622	116.822	217.547
Arts / Museum	Non-Profit	2212	0.503	14.089	52.970	190.470
Arts / Museum	Theater	2214	0.398	16.109	79.098	200.400
Arts / Museum	Art Gallery	4781	0.444	15.116	64.091	148.243
Arts / Museum	Event Space	5249	0.425	16.340	73.055	200.883
City / Outdoors	Embassy	225	0.428	16.696	63.396	183.067
City / Outdoors	Skate Park	294	0.458	12.425	53.605	161.497
City / Outdoors	Campground	364	0.467	14.627	56.673	243.453
City / Outdoors	Outdoors & Recreation	407	0.500	13.380	57.111	171.027
City / Outdoors	Landmark	465	0.453	16.127	68.301	177.912
City / Outdoors	Construction	482	0.500	15.876	51.510	199.662
City / Outdoors	Sculpture	488	0.414	15.607	77.418	171.502
City / Outdoors	Lake	493	0.458	14.475	52.140	155.791
City / Outdoors	Military Base	529	0.493	15.830	56.692	223.726
City / Outdoors	Cemetery	562	0.471	14.528	60.135	157.552
City / Outdoors	Community Center	614	0.503	11.065	51.288	177.347
City / Outdoors	Bridge	649	0.469	15.009	61.430	197.881
City / Outdoors	Trail	1012	0.472	14.239	51.978	148.435
City / Outdoors	City	1078	0.476	12.645	60.536	162.048
City / Outdoors	Field	1120	0.465	12.430	55.157	167.885
City / Outdoors	City Hall	1181	0.491	12.819	64.788	238.043
City / Outdoors	Garden	1211	0.453	14.984	61.143	169.182
City / Outdoors	Courthouse	1293	0.423	16.928	102.279	339.494
City / Outdoors	Historic Site	1354	0.442	15.873	63.486	166.154
City / Outdoors	Scenic Lookout	1362	0.445	15.014	60.848	154.530
City / Outdoors	Beach	1439	0.408	15.134	66.939	178.170
City / Outdoors	Plaza	1457	0.415	15.199	82.700	177.887
City / Outdoors	Dog Run	1511	0.465	12.711	50.760	151.284
City / Outdoors	Housing Development	2822	0.527	12.362	40.505	158.843
City / Outdoors	Neighborhood	3302	0.511	12.372	53.700	150.419
City / Outdoors	Other Outdoors	3386	0.461	13.403	59.373	160.382
City / Outdoors	Playground	3582	0.478	11.743	59.091	153.390

Supplementary Table 2: Summary statistics of the venues categories used in our models grouped by types of places. Only categories with more than 200 venues are shown. Source data are provided as a Source Data file. (continued)

Taxonomy	Category	Number of venues	Avg. Income Segregation	Average Distance	Average # of users	Average # of stays
City / Outdoors	Government	5662	0.467	15.533	78.812	287.328
City / Outdoors	Park	6214	0.474	12.009	52.844	152.693
City / Outdoors	Residential	23696	0.504	13.174	47.564	169.774
Coffee / Tea	Tea Room	531	0.381	15.151	92.111	169.979
Coffee / Tea	Bubble Tea	568	0.395	12.786	87.891	141.741
Coffee / Tea	Cafeteria	1458	0.442	15.074	78.431	318.456
Coffee / Tea	Coffee Shop	9870	0.391	12.743	127.712	277.431
College	Sorority House	203	0.523	13.004	40.443	128.517
College	IT Services	214	0.445	17.382	58.290	219.561
College	Communications	228	0.442	13.396	55.053	174.513
College	College & Education	255	0.452	12.485	56.141	194.075
College	Technology	300	0.422	15.182	64.163	248.973
College	Rec Center	366	0.460	12.754	56.699	196.355
College	Recreation Center	390	0.450	12.442	80.023	268.256
College	College Bookstore	466	0.443	13.303	59.491	161.122
College	Quad	471	0.443	13.871	62.255	152.062
College	Medical School	521	0.418	16.263	76.106	283.856
College	Frat House	652	0.512	13.248	49.391	141.701
College	Science	724	0.434	12.737	60.521	242.238
College	Community College	732	0.425	13.573	62.495	222.971
College	Auditorium	932	0.427	15.136	68.609	239.104
College	University	1119	0.440	15.631	59.177	210.475
College	Residence Hall	1133	0.530	13.810	42.969	147.200
College	Trade School	1298	0.450	16.425	59.787	231.831
College	Lab	1331	0.432	14.421	55.772	191.867
College	Student Center	1882	0.481	12.528	55.139	183.011
College	Administrative Building	2040	0.459	14.540	55.263	194.597
College	Academic Building	2974	0.449	13.510	58.359	217.623
College	Library	3826	0.470	10.788	73.320	226.029
College	Classroom	3838	0.437	12.787	52.975	182.332
Entertainment	Beer Bar	206	0.337	14.858	117.898	206.461
Entertainment	Gaming Cafe	215	0.461	12.819	58.837	133.451
Entertainment	Water Park	233	0.424	15.610	71.554	208.824
Entertainment	Surf Spot	289	0.419	15.460	69.554	176.433
Entertainment	Cineplex	370	0.340	12.650	241.438	456.343
Entertainment	Casino	422	0.425	16.977	148.270	523.559
Entertainment	Social Club	479	0.451	11.788	62.084	221.956
Entertainment	Winery	550	0.408	17.228	79.436	159.800
Entertainment	Resort	733	0.435	17.815	59.608	196.113
Entertainment	Karaoke	782	0.427	13.220	70.532	165.822
Entertainment	Hookah Bar	827	0.457	12.424	54.873	134.173
Entertainment	Theme Park	1021	0.336	25.889	174.495	271.523
Entertainment	Arcade	1316	0.409	15.056	123.954	254.209
Entertainment	Brewery	1354	0.391	14.781	97.516	223.493
Entertainment	Wine Bar	1574	0.441	13.844	67.503	150.335
Entertainment	Cocktail	1608	0.399	15.540	84.113	182.987
Entertainment	Speakeasy	1747	0.473	12.902	46.722	134.085
Entertainment	Dive Bar	1761	0.448	11.773	62.341	173.660
Entertainment	Pub	1905	0.384	12.802	97.048	217.520
Entertainment	Sports Bar	1931	0.376	14.300	131.401	305.056

Supplementary Table 2: Summary statistics of the venues categories used in our models grouped by types of places. Only categories with more than 200 venues are shown. Source data are provided as a Source Data file. (continued)

Taxonomy	Category	Number of venues	Avg. Income Segregation	Average Distance	Average # of users	Average # of stays
Entertainment	Nightlife	2134	0.490	13.843	59.323	173.229
Entertainment	Dance Studio	2191	0.450	12.551	51.251	132.293
Entertainment	Nightclub	2243	0.450	15.428	76.845	192.681
Entertainment	Smoke Shop	2482	0.443	12.279	61.069	133.351
Entertainment	Pool	5584	0.468	12.456	52.940	190.044
Entertainment	Spa	5648	0.426	12.216	69.536	154.326
Entertainment	Entertainment	6846	0.417	16.488	81.195	197.295
Entertainment	Bar	10471	0.431	13.426	85.961	210.751
Food	African	207	0.533	11.947	71.169	172.783
Food	Eastern European	207	0.449	12.007	71.271	150.256
Food	Health Food Store	239	0.417	11.512	75.854	150.464
Food	Filipino	246	0.417	12.896	71.211	138.496
Food	Bistro	251	0.389	14.589	90.355	193.884
Food	Tapas	280	0.376	14.221	82.511	152.786
Food	Ramen	289	0.341	12.928	88.682	142.512
Food	Brazilian	294	0.438	14.288	99.187	201.653
Food	Hawaiian	312	0.367	12.989	96.532	158.205
Food	Smoothie Shop	315	0.358	11.880	112.311	242.952
Food	Tex-Mex	364	0.327	12.720	341.228	601.047
Food	Cajun / Creole	379	0.400	14.828	113.934	211.741
Food	Cupcakes	383	0.441	13.437	75.123	147.138
Food	Peruvian	410	0.417	11.563	78.741	146.322
Food	Noodles	418	0.348	13.509	106.732	179.608
Food	South American	418	0.480	11.569	75.026	157.177
Food	Burritos	443	0.411	13.277	86.332	165.569
Food	Spanish	561	0.552	10.379	67.547	158.701
Food	Southern / Soul	652	0.528	12.458	89.206	180.278
Food	Vegetarian / Vegan	655	0.412	13.445	78.725	163.765
Food	Salad	658	0.348	15.133	113.520	232.347
Food	Farm	662	0.425	17.896	76.302	183.054
Food	Gastropub	667	0.344	13.780	109.087	208.927
Food	Cuban	733	0.446	12.534	109.518	234.900
Food	French	775	0.367	14.872	82.875	169.714
Food	Middle Eastern	881	0.414	12.928	75.260	146.427
Food	Candy Store	887	0.442	16.061	79.665	153.422
Food	Food Court	906	0.359	14.796	208.423	510.752
Food	Yogurt	969	0.376	12.399	96.181	161.340
Food	Greek	973	0.391	13.418	93.135	180.267
Food	Beer Garden	1018	0.434	14.610	72.674	174.102
Food	Hot Dogs	1063	0.418	14.320	145.469	274.330
Food	Wings	1136	0.406	12.861	175.960	317.189
Food	Food & Drink	1137	0.461	12.177	73.593	180.035
Food	New American	1214	0.350	15.918	114.366	222.959
Food	Snacks	1252	0.411	15.701	129.819	279.585
Food	Caribbean	1321	0.547	10.387	73.995	161.611
Food	Mediterranean	1389	0.375	13.748	88.261	165.324
Food	Juice Bar	1406	0.408	12.794	92.651	189.915
Food	Tacos	1479	0.482	12.893	104.023	208.667
Food	Korean	1560	0.386	12.642	91.701	172.747
Food	Latin American	1588	0.509	10.887	90.205	187.176

Supplementary Table 2: Summary statistics of the venues categories used in our models grouped by types of places. Only categories with more than 200 venues are shown. Source data are provided as a Source Data file. (continued)

Taxonomy	Category	Number of venues	Avg. Income Segregation	Average Distance	Average # of users	Average # of stays
Food	Steakhouse	1628	0.336	14.946	175.693	356.500
Food	Fried Chicken	1682	0.518	12.370	101.608	192.762
Food	Vietnamese	1692	0.378	11.979	79.474	138.682
Food	Indian	1769	0.393	13.172	66.745	125.743
Food	Desserts	1925	0.418	13.874	89.380	152.774
Food	Bagels	1930	0.424	11.992	67.607	133.167
Food	Thai	2193	0.395	12.218	71.367	126.502
Food	Food	2208	0.516	11.997	60.094	164.189
Food	BBQ	2323	0.429	14.098	106.632	211.879
Food	Japanese	2448	0.369	13.321	89.347	156.733
Food	Breakfast	2824	0.399	12.248	127.996	246.725
Food	Sushi	3072	0.377	12.626	83.351	147.456
Food	Seafood	3130	0.419	14.191	127.042	252.414
Food	Food Truck	3375	0.453	15.008	75.165	204.978
Food	Asian	3441	0.414	12.823	85.262	157.514
Food	Lounge	3779	0.447	16.673	80.957	205.969
Food	Burgers	4050	0.389	13.315	147.640	259.979
Food	Restaurant	4308	0.419	13.903	103.671	226.953
Food	Ice Cream	4572	0.423	12.337	80.044	142.181
Food	Deli / Bodega	5143	0.489	13.142	63.030	160.093
Food	Donuts	5218	0.459	12.037	80.235	165.157
Food	Bakery	5678	0.453	12.283	85.886	177.006
Food	Café	5860	0.411	14.912	86.765	234.605
Food	Italian	6974	0.382	13.580	95.772	187.551
Food	Chinese	8141	0.453	11.555	77.170	145.636
Food	Sandwiches	9402	0.422	13.211	103.663	214.742
Food	American	10497	0.384	14.371	134.053	286.301
Food	Mexican	10891	0.443	12.322	108.892	213.131
Food	Fast Food	12542	0.436	12.434	159.636	296.520
Food	Pizza	14476	0.452	11.671	79.331	165.050
Grocery	Butcher	555	0.486	11.023	79.168	177.256
Grocery	Wine Shop	936	0.415	12.743	88.110	175.282
Grocery	Market	1027	0.479	10.305	108.668	263.747
Grocery	Farmer's Market	1247	0.448	12.170	74.882	163.005
Grocery	Supermarket	2346	0.431	8.899	282.016	828.129
Grocery	Liquor Store	5455	0.488	10.861	65.787	140.822
Grocery	Grocery Store	7500	0.462	8.966	200.073	536.864
Grocery	Convenience Store	7777	0.497	12.532	78.048	180.765
Health	Medical Facility (I)	387	0.349	15.861	121.711	423.685
Health	Medical Facility (II)	531	0.465	14.363	50.650	162.533
Health	Medical Facility (III)	555	0.442	12.701	48.602	123.769
Health	Medical Facility (IV)	891	0.406	12.622	77.132	152.237
Health	Medical Facility (V)	1092	0.385	15.029	136.079	392.520
Health	Medical Facility (VI)	1366	0.409	12.976	95.669	198.462
Health	Medical Facility (VII)	1372	0.439	12.646	56.843	144.797
Health	Medical Facility (VII)	1611	0.423	13.994	66.801	173.552
Health	Medical Facility (IX)	1622	0.507	13.716	49.383	293.327
Health	Medical Facility (X)	3509	0.443	12.070	50.654	124.608
Health	Medical Facility (XI)	5348	0.384	15.840	114.005	412.862
Health	Medical Facility (XII)	10770	0.447	14.140	66.931	215.758

Supplementary Table 2: Summary statistics of the venues categories used in our models grouped by types of places. Only categories with more than 200 venues are shown. Source data are provided as a Source Data file. (continued)

Taxonomy	Category	Number of venues	Avg. Income Segregation	Average Distance	Average # of users	Average # of stays
Health	Medical Facility (XIII)	15838	0.451	12.002	53.035	120.416
Health	Medical Facility (XIV)	28508	0.426	13.454	62.408	148.640
Religious	Kingdom Hall	206	0.570	9.214	45.573	264.150
Religious	Mosque	384	0.532	10.084	52.297	236.763
Religious	Temple	593	0.485	13.526	50.936	168.624
Religious	Synagogue	819	0.511	9.599	50.994	208.485
Religious	Spiritual	1166	0.480	13.514	51.088	186.915
Religious	Church	17333	0.499	10.731	54.555	227.610
Education	Education (I)	372	0.461	11.802	47.798	140.801
Education	Education (II)	485	0.507	11.669	42.645	154.678
Education	Education (III)	843	0.540	10.731	43.791	159.936
Education	Education (IV)	1569	0.523	9.701	58.000	315.715
Education	Education (V)	2101	0.461	14.319	55.636	199.006
Education	Education (VI)	4379	0.503	10.061	69.680	403.168
Education	Education (VII)	5642	0.554	9.314	44.459	233.523
Education	Education (VIII)	6993	0.513	10.914	48.173	197.022
Service	Rental Service	228	0.498	13.032	105.110	292.057
Service	Radio Station	242	0.446	15.941	62.979	203.975
Service	Hostel	243	0.487	13.064	45.922	127.230
Service	Check Cashing Service	267	0.624	9.293	62.015	128.071
Service	Travel Agency	287	0.440	13.832	62.411	151.742
Service	Home Services	312	0.461	13.706	103.731	309.606
Service	Other Repair	369	0.429	12.999	75.691	166.528
Service	Tailor	376	0.447	13.604	53.572	120.960
Service	Indie Movies	382	0.394	13.769	89.455	191.089
Service	Animal Shelter	388	0.476	13.314	57.758	185.162
Service	Weight Loss Center	404	0.400	10.794	74.584	146.559
Service	Auto Garage	409	0.498	14.264	55.462	187.927
Service	Recycling	414	0.506	12.490	60.626	195.971
Service	Garden Center	431	0.477	12.226	82.877	223.088
Service	Pilates Studio	440	0.397	11.758	55.905	115.355
Service	Gymnastics Gym	477	0.432	12.525	63.082	227.178
Service	Laundromat	503	0.584	9.002	55.467	143.499
Service	B & B	559	0.458	14.710	43.725	132.698
Service	Insurance Office	570	0.478	13.116	54.333	149.714
Service	Gourmet	591	0.417	13.850	76.929	165.030
Service	Bridal	652	0.421	14.272	62.512	125.494
Service	ATM	679	0.435	10.782	97.427	202.769
Service	Dry Cleaner	726	0.448	11.386	57.605	115.751
Service	Hotel Bar	867	0.372	19.143	76.543	193.978
Service	Business Services	907	0.445	14.629	68.816	217.485
Service	Pet Service	1100	0.469	12.121	49.037	127.552
Service	Real Estate	1144	0.457	13.133	49.453	143.086
Service	Motel	1165	0.495	14.730	57.947	200.490
Service	Lawyer	1205	0.453	15.450	54.998	184.332
Service	Massage Studio	1271	0.416	12.410	59.540	126.324
Service	Health & Beauty	1357	0.424	11.876	68.405	139.170
Service	Tanning Salon	1478	0.419	10.749	63.576	128.497
Service	Tattoo	1517	0.481	12.626	50.339	114.987
Service	Funeral Home	1577	0.445	13.706	62.995	155.798

Supplementary Table 2: Summary statistics of the venues categories used in our models grouped by types of places. Only categories with more than 200 venues are shown. Source data are provided as a Source Data file. (continued)

Taxonomy	Category	Number of venues	Avg. Income Segregation	Average Distance	Average # of users	Average # of stays
Service	Design	1727	0.458	14.922	49.466	149.559
Service	Shipping Store	1837	0.418	13.630	77.607	183.259
Service	Yoga Studio	1839	0.422	12.133	56.229	130.999
Service	Credit Union	1851	0.443	14.357	76.355	209.182
Service	Storage	1897	0.510	13.515	50.424	159.418
Service	Martial Arts	2281	0.445	11.428	53.611	129.340
Service	Optical	2464	0.421	11.386	119.599	256.223
Service	Car Washes	3009	0.489	10.821	82.476	188.508
Service	Rental Car	3036	0.471	15.272	63.001	183.241
Service	Veterinarians	3167	0.467	11.992	59.867	141.594
Service	Pet Store	3540	0.426	11.235	85.082	161.507
Service	Post Office	3573	0.493	12.878	74.065	218.873
Service	Financial / Legal	4532	0.475	12.374	69.492	173.768
Service	Auto Dealer	5072	0.492	15.993	87.589	378.012
Service	Hotel	6917	0.421	18.898	66.722	212.857
Service	Nail Salon	7249	0.451	10.732	65.269	128.488
Service	Gym / Fitness	7774	0.423	10.849	79.472	300.069
Service	Laundry	8645	0.490	9.946	56.002	129.049
Service	Pharmacy	8916	0.456	9.722	128.197	266.970
Service	Gym	10942	0.443	12.154	62.133	228.885
Service	Bank	15567	0.457	11.468	80.154	179.052
Service	Gas Station	18280	0.500	12.945	84.420	186.757
Service	Automotive	23480	0.514	12.741	59.779	178.267
Service	Salon / Barbershop	25162	0.470	11.055	62.901	136.973
Shopping	Cycle Studio	220	0.364	14.354	73.982	174.982
Shopping	Newsstand	243	0.374	22.980	119.333	249.333
Shopping	Motorcycle Shop	299	0.460	15.756	66.542	195.405
Shopping	Warehouse Store	352	0.371	11.369	734.801	2486.142
Shopping	Board Shop	362	0.437	13.164	62.787	120.657
Shopping	Record Shop	402	0.468	13.027	63.933	130.062
Shopping	Print Shop	406	0.481	14.634	73.958	192.855
Shopping	Shopping Plaza	459	0.391	11.256	98.142	170.429
Shopping	Flea Market	489	0.470	14.583	109.384	265.650
Shopping	Mattress Store	565	0.422	11.896	89.361	185.731
Shopping	Lingerie	577	0.381	14.751	119.660	181.797
Shopping	Shops	928	0.463	12.725	64.823	152.456
Shopping	Music Store	967	0.441	13.220	67.580	146.850
Shopping	Supplement Shop	997	0.399	11.159	91.205	154.033
Shopping	Hobbies	1041	0.429	13.573	65.980	141.393
Shopping	Accessories	1092	0.367	15.886	118.582	183.751
Shopping	Video Games	1121	0.423	11.156	104.806	159.447
Shopping	Kids Store	1135	0.388	13.454	96.496	151.435
Shopping	Antiques	1179	0.423	14.472	55.559	117.534
Shopping	Toys & Games	1213	0.378	13.933	120.670	197.754
Shopping	Men's Store	1231	0.414	15.100	86.097	155.247
Shopping	Bike Shop	1236	0.457	12.600	55.547	130.321
Shopping	Big Box Store	1272	0.362	9.825	776.689	2030.443
Shopping	Office Supplies	1507	0.423	11.919	113.760	228.774
Shopping	Bookstore	1553	0.400	14.066	105.184	187.956
Shopping	Flower Shop	1881	0.461	12.462	54.217	132.182

Supplementary Table 2: Summary statistics of the venues categories used in our models grouped by types of places. Only categories with more than 200 venues are shown. Source data are provided as a Source Data file. (continued)

Taxonomy	Category	Number of venues	Avg. Income Segregation	Average Distance	Average # of users	Average # of stays
Shopping	Thrift / Vintage	2140	0.443	11.407	86.736	194.530
Shopping	Boutique	2448	0.409	14.564	68.438	123.245
Shopping	Jewelry	2947	0.397	14.173	81.253	140.516
Shopping	Electronics	3052	0.438	13.671	144.828	288.213
Shopping	Mall	3082	0.390	12.304	118.657	204.918
Shopping	Women's Store	3118	0.394	13.484	96.509	151.126
Shopping	Department Store	3152	0.374	12.038	356.501	764.550
Shopping	Sporting Goods	3159	0.399	14.722	134.976	242.046
Shopping	Video Store	3315	0.454	10.366	87.291	172.770
Shopping	Discount Store	3470	0.491	9.418	144.520	267.148
Shopping	Shoes	3586	0.424	13.037	104.711	167.757
Shopping	Mobile Phones	3885	0.450	11.858	92.191	171.337
Shopping	Hardware	5543	0.454	11.764	211.612	614.387
Shopping	Furniture / Home	5800	0.417	13.824	121.082	237.950
Shopping	Apparel	6827	0.392	14.201	145.037	234.697
Shopping	Cosmetics	7793	0.443	11.850	84.273	154.297
Shopping	Shop	9284	0.461	12.782	78.512	176.747
Sports	Baseball	369	0.350	19.823	70.453	162.539
Sports	Racetrack	385	0.371	23.164	87.878	234.286
Sports	Stadium	386	0.351	21.214	136.705	304.482
Sports	Billiards	434	0.440	11.649	69.152	181.270
Sports	Skating Rink	480	0.356	17.048	122.417	378.435
Sports	Football	630	0.372	20.860	126.892	326.852
Sports	Tennis Court	785	0.463	11.538	50.805	181.855
Sports	Basketball Court	797	0.464	13.474	73.339	245.417
Sports	Bowling Alley	890	0.362	13.177	132.151	418.672
Sports	Soccer Field	987	0.431	13.354	65.214	189.379
Sports	Golf Course	1544	0.414	13.189	83.422	312.324
Sports	Baseball Field	1864	0.444	13.227	52.533	161.803
Sports	Athletics & Sports	2540	0.438	14.604	68.404	199.065
Transportation	Transportation Services	226	0.474	16.283	62.159	253.044
Transportation	Airport	286	0.422	20.566	85.941	287.706
Transportation	Platform	290	0.374	25.441	129.883	255.066
Transportation	Track	383	0.430	14.194	60.321	175.491
Transportation	Rest Areas	518	0.455	15.582	70.199	185.176
Transportation	Terminal	651	0.398	23.224	176.469	399.373
Transportation	Light Rail	703	0.483	15.665	74.296	237.459
Transportation	Metro	780	0.511	13.652	85.042	187.344
Transportation	Bike	789	0.436	16.035	68.511	164.631
Transportation	Plane	813	0.391	21.042	74.250	174.474
Transportation	Train Station	979	0.451	16.933	69.431	177.780
Transportation	Boat / Ferry	1124	0.408	17.243	65.294	196.523
Transportation	Train	1181	0.475	17.195	59.510	169.062
Transportation	Harbor / Marina	1291	0.431	14.771	65.112	230.017
Transportation	Gate	1294	0.310	24.742	162.175	308.270
Transportation	Taxi	1461	0.452	16.255	57.915	162.012
Transportation	Bus Stop	2328	0.470	13.091	58.416	135.324
Transportation	Travel	2815	0.467	15.486	67.037	179.067
Transportation	Intersection	3052	0.469	12.195	67.253	152.627
Transportation	Gift Shop	3094	0.376	16.311	93.598	169.777

Supplementary Table 2: Summary statistics of the venues categories used in our models grouped by types of places. Only categories with more than 200 venues are shown. Source data are provided as a Source Data file. (continued)

Taxonomy	Category	Number of venues	Avg. Income Segregation	Average Distance	Average # of users	Average # of stays
Transportation	Parking	4650	0.411	16.840	85.148	214.203
Transportation	Bus Station	4766	0.480	14.377	63.051	157.623
Transportation	Bus	7374	0.498	13.359	57.988	146.324
Transportation	Road	11687	0.470	12.744	59.350	145.178
Work	Campaign	211	0.461	13.366	54.716	145.801
Work	Business Center	391	0.442	18.280	60.074	256.384
Work	Advertising Agency	399	0.425	18.169	61.053	214.524
Work	Distributor	415	0.530	17.036	52.224	424.166
Work	Warehouse	449	0.552	17.655	48.176	346.619
Work	Convention Center	643	0.350	24.241	129.303	299.507
Work	Meeting Room	883	0.390	19.322	82.353	234.553
Work	Conference room	1796	0.446	17.145	58.384	224.545
Work	Factory	2739	0.538	16.944	45.292	388.777
Work	Professional	3232	0.502	14.158	51.488	197.478
Work	Tech Startup	3252	0.445	16.046	52.117	194.531
Work	Coworking Space	6099	0.463	16.838	57.650	244.066
Work	Building	28773	0.486	15.794	55.432	235.989
Work	Office	39508	0.456	17.291	53.982	244.368

Supplementary Table 3: Regression results of the OLS models for place income segregation by metro area. Bars each variable is the relative importance (in %) of that variable in the metro area using the LMG method [13]. Source data are provided as a Source Data file.

	Dependent variable:											
	Place income segregation by metro area											
	Boston	Chicago	Chicago*	Dallas	Detroit	LA	Miami	New York	Philadelphia	SF	Seattle	Washington
Catchment range	−0.243***	−0.238***	−0.308***	−0.246***	−0.239***	−0.201***	−0.203***	−0.264***	−0.252***	−0.184***	−0.195***	−0.247***
Area income	−0.066***	−0.074***	−0.075***	−0.067***	−0.068***	−0.041***	−0.101***	−0.069***	−0.070***	−0.062***	0.015**	−0.029***
# Neighbors	−0.119***	−0.138***	−0.072***	−0.172***	−0.135***	−0.141***	−0.208***	−0.117***	−0.129***	−0.152***	−0.141***	−0.140***
Price tier			−0.034***									
Rating			−0.088***									
Constant	−0.257**	−0.023	−0.076	0.239***	0.099*	−0.156***	−0.578***	−0.620***	−0.250***	−0.218**	−0.055	−0.299***
	(0.114)	(0.051)	(0.081)	(0.084)	(0.060)	(0.058)	(0.067)	(0.037)	(0.052)	(0.087)	(0.098)	(0.084)
Category effect	YES	YES	YES	YES	YES	YES	YES	YES	YES	YES	YES	YES
PUMA area fixed effect	YES	YES	YES	YES	YES	YES	YES	YES	YES	YES	YES	YES
Observations	33,888	127,521	14,096	93,775	48,398	156,728	85,027	171,759	58,160	47,444	36,772	67,553
R^2	0.216	0.288	0.362	0.205	0.241	0.212	0.204	0.294	0.350	0.180	0.158	0.239
Adjusted R^2	0.209	0.285	0.355	0.202	0.236	0.209	0.200	0.292	0.346	0.174	0.151	0.235

Note: 0%, 25%, 50%, 75%, (two-sided) $^{*}p<0.1$; $^{**}p<0.05$; $^{***}p<0.01$

Supplementary Table 4: Regression table for the different models of individual income segregation S_i. Models are constructed including different groups of variables. Only variables with p-value < 0.01 in the full model are shown. Fixed factors are not show either. For places we only show the top 10 with largest positive and negative coefficients. Source data are provided as a Source Data file.

		Models for Individual Income Segregation S_i						
Group	Variable	$\{R_i\}$	$\{M_i\}$	$\{P_i\}$	$\{R_i, M_i\}$	$\{R_i, P_i\}$	$\{M_i, P_i\}$	$\{R_i, M_i, P_i\}$
Residential	Bachelor degree or more	-0.0060 ***			-0.0060 ***	-0.0028 ***		-0.0032 ***
	Below 9th grade	0.0112 ***			0.0107 ***	0.0079 ***		0.0077 ***
	From 9th to 12th grade	0.0075 ***			0.0069 ***	0.0048 ***		0.0046 ***
	Employed civilian	-0.0069 ***			-0.0069 ***	-0.0064 ***		-0.0065 ***
	Total labor force	0.0047 ***			0.0047 ***	0.0035 ***		0.0036 ***
	Agriculture	0.0002			0.0007 ***	0.0002		0.0005 ***
	Arts	-0.0021 ***			-0.0020 ***	-0.0018 ***		-0.0018 ***
	Construction	-0.0009 ***			-0.0005 **	-0.0009 ***		-0.0007 ***
	Finance	-0.0038 ***			-0.0036 ***	-0.0029 ***		-0.0029 ***
	Information	-0.0025 ***			-0.0024 ***	-0.0020 ***		-0.0020 ***
	Professional	-0.0043 ***			-0.0042 ***	-0.0037 ***		-0.0037 ***
	Public	-0.0037 ***			-0.0031 ***	-0.0032 ***		-0.0030 ***
	Retail	-0.0009 ***			-0.0009 ***	-0.0009 ***		-0.0009 ***
	Transportation	-0.0013 ***			-0.0013 ***	-0.0010 ***		-0.0010 ***
	Asian	-0.0032 ***			-0.0021 ***	-0.0023 ***		-0.0017 ***
	Black	0.0060 ***			0.0065 ***	0.0048 ***		0.0053 ***
	White	-0.0094 ***			-0.0081 ***	-0.0064 ***		-0.0058 ***
	Car	-0.0053 ***			-0.0048 ***	-0.0061 ***		-0.0056 ***
	Public transportation	0.0010 **			0.0012 **	0.0011 **		0.0012 ***
	Median income	0.0082 ***			0.0082 ***	0.0093 ***		0.0095 ***
	Poverty ratio	0.0074 ***			0.0077 ***	0.0064 ***		0.0065 ***
Mobility	Distance from home		-0.0020 ***		-0.0012 ***		-0.0101 ***	-0.0069 ***
	Radius of gyration		-0.0388 ***		-0.0391 ***		-0.0141 ***	-0.0153 ***
Places	Coffee Shop			-0.0114 ***		-0.0086 ***	-0.0114 ***	-0.0083 ***
	Office			-0.0118 ***		-0.0091 ***	-0.0107 ***	-0.0077 ***
	Gym / Fitness			-0.0073 ***		-0.0057 ***	-0.0073 ***	-0.0056 ***
	American			-0.0088 ***		-0.0060 ***	-0.0078 ***	-0.0055 ***
	Gym			-0.0077 ***		-0.0048 ***	-0.0076 ***	-0.0046 ***
	Café			-0.0053 ***		-0.0047 ***	-0.0052 ***	-0.0044 ***
	Parking			-0.0039 ***		-0.0046 ***	-0.0037 ***	-0.0042 ***
	Coworking space			-0.0047 ***		-0.0043 ***	-0.0043 ***	-0.0039 ***
	Gift shop			-0.0048 ***		-0.0040 ***	-0.0045 ***	-0.0039 ***
	Italian			-0.0069 ***		-0.0039 ***	-0.0066 ***	-0.0038 ***
	Spanish			0.0036 ***		0.0019 ***	0.0034 ***	0.0018 ***
	Check cashing			0.0042 ***		0.0021 ***	0.0040 ***	0.0020 ***
	Discount store			0.0062 ***		0.0024 ***	0.0056 ***	0.0020 ***
	Laundromat			0.0050 ***		0.0022 ***	0.0047 ***	0.0022 ***
	Automotive			0.0042 ***		0.0018 ***	0.0047 ***	0.0023 ***
	Residence hall			0.0037 ***		0.0024 ***	0.0037 ***	0.0024 ***
	Southern / soul			0.0055 ***		0.0025 ***	0.0054 ***	0.0025 ***
	Education (VII)			0.0049 ***		0.0029 ***	0.0043 ***	0.0026 ***
	Caribbean			0.0055 ***		0.0029 ***	0.0052 ***	0.0029 ***
	Latin American			0.0044 ***		0.0031 ***	0.0040 ***	0.0031 ***
Fixed effects	PUMA area	YES	NO	NO	YES	YES	NO	YES
Observations		1037706	1037706	1037706	1037706	1037706	1037706	1037706
R^2		0.256	0.057	0.269	0.304	0.424	0.285	0.435
MSE		0.021	0.027	0.021	0.02	0.016	0.02	0.016
Note:						(two-sided) *p<0.1; **p<0.05; ***p<0.01		

Supplementary Table 5: Regression table for the different models of individual social exploration $\sigma_{s,i}$. Models are constructed including different groups of variables. Only variables with p-value < 0.01 in the full model are shown. Fixed factors are not show either. For places we only show the top 10 with largest positive and negative coefficients. Source data are provided as a Source Data file.

		Models for Individual Social Exploration $\sigma_{s,i}$						
Group	Variable	$\{R_i\}$	$\{M_i\}$	$\{P_i\}$	$\{R_i, M_i\}$	$\{R_i, P_i\}$	$\{M_i, P_i\}$	$\{R_i, M_i, P_i\}$
Residential	Bachelor degree or more	-0.0006 *			0.0001	-0.0024 ***		-0.0013 ***
	Below 9th grade	-0.0102 ***			-0.0097 ***	-0.0080 ***		-0.0076 ***
	From 9th to 12th grade	-0.0056 ***			-0.0054 ***	-0.0040 ***		-0.0039 ***
	Employment civilian	0.0124 ***			0.0125 ***	0.0114 ***		0.0117 ***
	Total labor force	-0.0073 ***			-0.0075 ***	-0.0065 ***		-0.0066 ***
	Agriculture	-0.0005 ***			-0.0009 ***	-0.0005 ***		-0.0008 ***
	Arts	0.0015 ***			0.0017 ***	0.0012 ***		0.0015 ***
	Construction	-0.0017 ***			-0.0021 ***	-0.0017 ***		-0.0020 ***
	Information	0.0014 ***			0.0015 ***	0.0010 ***		0.0011 ***
	Manufacturing	-0.0031 ***			-0.0032 ***	-0.0033 ***		-0.0033 ***
	Professional	0.0012 ***			0.0013 ***	0.0007 **		0.0009 ***
	Public	0.0025 ***			0.0023 ***	0.0021 ***		0.0020 ***
	Retail	-0.0006 ***			-0.0005 **	-0.0008 ***		-0.0006 ***
	Wholesale	-0.0011 ***			-0.0011 ***	-0.0012 ***		-0.0012 ***
	Black	-0.0073 ***			-0.0081 ***	-0.0066 ***		-0.0073 ***
	White	0.0039 ***			0.0032 ***	0.0021 ***		0.0015 ***
	Car	0.0093 ***			0.0088 ***	0.0095 ***		0.0088 ***
	Public transportation	-0.0068 ***			-0.0069 ***	-0.0071 ***		-0.0071 ***
	Walked	-0.0010 ***			-0.0007 ***	-0.0013 ***		-0.0007 ***
	Median income	-0.0191 ***			-0.0198 ***	-0.0189 ***		-0.0196 ***
	Poverty ratio	-0.0136 ***			-0.0136 ***	-0.0128 ***		-0.0127 ***
Mobility	Radius of gyration		0.0074 ***		0.0049 ***		-0.0031 ***	-0.0023 ***
	Distance from home		0.0215 ***		0.0179 ***		0.0234 ***	0.0188 ***
Places	Gas station			-0.0051 ***		-0.0032 ***	-0.0058 ***	-0.0033 ***
	Discount store			-0.0079 ***		-0.0036 ***	-0.0073 ***	-0.0031 ***
	Laundry			-0.0063 ***		-0.0031 ***	-0.0058 ***	-0.0029 ***
	Education (VII)			-0.0054 ***		-0.0031 ***	-0.0048 ***	-0.0027 ***
	Residence hall			-0.0045 ***		-0.0027 ***	-0.0044 ***	-0.0027 ***
	Food			-0.0063 ***		-0.0026 ***	-0.0062 ***	-0.0025 ***
	Convenience Store			-0.0044 ***		-0.0023 ***	-0.0047 ***	-0.0023 ***
	Check cashing			-0.0051 ***		-0.0022 ***	-0.0049 ***	-0.0022 ***
	Flea market			-0.0039 ***		-0.0022 ***	-0.0038 ***	-0.0021 ***
	Laundromat			-0.0055 ***		-0.0021 ***	-0.0052 ***	-0.0021 ***
	Bar			0.0014 ***		0.0023 ***	0.0013 ***	0.0022 ***
	Gym / fitness			0.0043 ***		0.0024 ***	0.0044 ***	0.0024 ***
	Office			0.0058 ***		0.0038 ***	0.0046 ***	0.0025 ***
	Italian			0.0063 ***		0.0025 ***	0.0063 ***	0.0026 ***
	Supermarket			0.0012 ***		0.0026 ***	0.0015 ***	0.0028 ***
	Warehouse Store			0.0041 ***		0.0030 ***	0.0041 ***	0.0030 ***
	Big Box Store			0.0050 ***		0.0036 ***	0.0048 ***	0.0038 ***
	Department store			0.0051 ***		0.0038 ***	0.0052 ***	0.0039 ***
	American			0.0078 ***		0.0042 ***	0.0072 ***	0.0042 ***
	Coffee shop			0.0079 ***		0.0047 ***	0.0080 ***	0.0045 ***
Fixed effects	PUMA area	YES	NO	NO	YES	YES	NO	YES
Observations		1037706	1037706	1037706	1037706	1037706	1037706	1037706
R^2		0.471	0.027	0.134	0.485	0.508	0.15	0.516
MSE		0.014	0.026	0.023	0.014	0.013	0.023	0.013
Note:					(two-sided) *p<0.1; **p<0.05; ***p<0.01			

Supplementary Table 6: Regression table for the different models of individual place exploration $\sigma_{p,i}$. Models are constructed including different groups of variables. Only variables with p-value < 0.01 in the full model are shown. Fixed factors are not show either. For places we only show the top 10 with largest positive and negative coefficients. Source data are provided as a Source Data file.

		Models for Individual Place exploration $\sigma_{p,i}$						
Group	Variable	$\{R_i\}$	$\{M_i\}$	$\{P_i\}$	$\{R_i, M_i\}$	$\{R_i, P_i\}$	$\{M_i, P_i\}$	$\{R_i, M_i, P_i\}$
Residential	Bachelor degree or more	0.0069 ***			0.0059 ***	0.0044 ***		0.0038 ***
	From 9th to 12th grade	-0.0024 ***			-0.0023 ***	-0.0008 ***		-0.0009 ***
	Public transportation	0.0011 **			0.0010 **	0.0012 ***		0.0011 ***
	Walked	-0.0008 ***			-0.0018 ***	-0.0011 ***		-0.0016 ***
	Median income	0.0037 ***			0.0045 ***	0.0027 ***		0.0032 ***
Mobility	Distance from home		-0.0238 ***		-0.0200 ***		-0.0119 ***	-0.0108 ***
	Radius of gyration		0.0448 ***		0.0495 ***		0.0228 ***	0.0249 ***
Places	Hardware			-0.0022 ***		-0.0022 ***	-0.0031 ***	-0.0030 ***
	Factory			-0.0021 ***		-0.0016 ***	-0.0024 ***	-0.0021 ***
	Distributor			-0.0010 ***		-0.0009 ***	-0.0012 ***	-0.0012 ***
	Casino			-0.0008 ***		-0.0008 ***	-0.0009 ***	-0.0011 ***
	Warehouse			-0.0009 ***		-0.0008 ***	-0.0010 ***	-0.0010 ***
	Motel			-0.0009 ***		-0.0005 ***	-0.0011 ***	-0.0008 ***
	Automotive			-0.0009 ***		-0.0002	-0.0012 ***	-0.0006 ***
	Building			-0.0008 ***		-0.0003 **	-0.0009 ***	-0.0006 ***
	Mosque			-0.0007 ***		-0.0006 ***	-0.0006 ***	-0.0006 ***
	Auto dealer			-0.0002 *		-0.0002 *	-0.0005 ***	-0.0005 ***
	Bank			0.0036 ***		0.0034 ***	0.0034 ***	0.0032 ***
	New american			0.0037 ***		0.0033 ***	0.0036 ***	0.0032 ***
	Parking			0.0034 ***		0.0034 ***	0.0034 ***	0.0033 ***
	Gas station			0.0037 ***		0.0044 ***	0.0028 ***	0.0035 ***
	Ice cream			0.0037 ***		0.0037 ***	0.0034 ***	0.0035 ***
	Video store			0.0036 ***		0.0038 ***	0.0035 ***	0.0037 ***
	Mall			0.0045 ***		0.0040 ***	0.0044 ***	0.0039 ***
	Womens store			0.0039 ***		0.0041 ***	0.0038 ***	0.0039 ***
	Coffee shop			0.0059 ***		0.0050 ***	0.0059 ***	0.0048 ***
	Pharmacy			0.0058 ***		0.0056 ***	0.0058 ***	0.0055 ***
Fixed effects	PUMA area	YES	NO	NO	YES	YES	NO	YES
Observations		1037706	1037706	1037706	1037706	1037706	1037706	1037706
R^2		0.037	0.061	0.284	0.109	0.292	0.298	0.307
MSE		0.019	0.018	0.014	0.017	0.014	0.014	0.013
Note:								(two-sided)*p<0.1; **p<0.05; ***p<0.01

Supplementary References

[1] Hariharan, R. & Toyama, K. Project lachesis: parsing and modeling location histories. In *International Conference on Geographic Information Science*, 106–124 (Springer, 2004).

[2] Cuttone, A., Lehmann, S. & Larsen, J. E. Inferring human mobility from sparse low accuracy mobile sensing data. In *Proceedings of the 2014 ACM International Joint Conference on Pervasive and Ubiquitous Computing: Adjunct Publication*, 995–1004 (2014).

[3] Ester, M., Kriegel, H.-P., Sander, J., Xu, X. *et al.* A density-based algorithm for discovering clusters in large spatial databases with noise. In *Kdd*, vol. 96, 226–231 (1996).

[4] U.S. Census Bureau. 2016 American Community Survey 5-Year Data. `https://www.census.gov/programs-surveys/acs` (2017). Accessed: 22-06-2019.

[5] Pew Research Center. Share of adults in the United States who owned a smartphone from 2011 to 2017, by location. (2017). URL `https://www.statista.com/statistics/195003/percentage-of-us-smartphone-owners-by-geographic-location/`. Accessed: 22-06-2019.

[6] Salganik, M. *Bit by bit: Social research in the digital age* (Princeton University Press, 2019).

[7] Jiang, S. *et al.* The timegeo modeling framework for urban mobility without travel surveys. *Proceedings of the National Academy of Sciences* **113**, E5370–E5378 (2016).

[8] Wang, Q., Phillips, N. E., Small, M. L. & Sampson, R. J. Urban mobility and neighborhood isolation in America's 50 largest cities. *Proceedings of the National Academy of Sciences* **115**, 7735–7740 (2018).

[9] Massey, D. S. & Denton, N. A. The dimensions of residential segregation. *Social forces* **67**, 281–315 (1988).

[10] Song, C., Koren, T., Wang, P. & Barabási, A.-L. Modelling the scaling properties of human mobility. *Nature Physics* **6**, 818 (2010).

[11] Pappalardo, L., Rinzivillo, S. & Simini, F. Human mobility modelling: exploration and preferential return meet the gravity model. *Procedia Computer Science* **83**, 934–939 (2016).

[12] Di Clemente, R. *et al.* Sequences of purchases in credit card data reveal lifestyles in urban populations. *Nature Communications* **9**, 3330 (2018).

[13] Lindeman, R. H., Merenda, P. F. & Gold, R. Z. *Introduction to bivariate and multivariate analysis* (Scott, Foresman & Co, Glenview, IL, 1980).

[14] R Core Team. *R: A Language and Environment for Statistical Computing*. R Foundation for Statistical Computing, Vienna, Austria (2020). URL `https://www.R-project.org/`.

[15] Dowle, M. & Srinivasan, A. *data.table: Extension of 'data.frame'* (2020). URL `https://CRAN.R-project.org/package=data.table`. R package version 1.13.4.

[16] LeDell, E. *et al.* *h2o: R Interface for the 'H2O' Scalable Machine Learning Platform* (2020). URL `https://CRAN.R-project.org/package=h2o`. R package version 3.30.0.1.

[17] Wickham, H. *ggplot2: Elegant Graphics for Data Analysis* (Springer-Verlag New York, 2016). URL `https://ggplot2.tidyverse.org`. R package version 3.3.2.

[18] Cheng, J., Karambelkar, B. & Xie, Y. *leaflet: Create Interactive Web Maps with the JavaScript 'Leaflet' Library* (2019). URL `https://CRAN.R-project.org/package=leaflet`. R package version 2.0.3.

[19] Walker, K. & Herman, M. *tidycensus: Load US Census Boundary and Attribute Data as 'tidyverse' and 'sf'-Ready Data Frames* (2020). URL `https://CRAN.R-project.org/package=tidycensus`. R package version 0.10.2.

[20] Walker, K. *tigris: Load Census TIGER/Line Shapefiles* (2020). URL `https://CRAN.R-project.org/package=tigris`. R package version 1.0.

[21] Hlavac, M. *stargazer: Well-Formatted Regression and Summary Statistics Tables*. Central European Labour Studies Institute (CELSI), Bratislava, Slovakia (2018). URL `https://CRAN.R-project.org/package=stargazer`. R package version 5.2.2.
