# Supplementary material for: Mobility patterns are associated with experienced income segregation in large US cities
Source: Nat Commun. 2021 Jul 30;12:4633. doi: 10.1038/s41467-021-24899-8 (PMC8324796; doi:10.1038/s41467-021-24899-8)
Supplement: Supplementary file 2 — Reporting Summary [file 41467_2021_24899_MOESM2_ESM.pdf]

nature research

Corresponding author(s): Esteban Moro

Last updated by author(s): Apr 19, 2021

Reporting Summary

Nature Research wishes to improve the reproducibility of the work that we publish. This form provides structure for consistency and transparency in reporting. For further information on Nature Research policies, see our Editorial Policies and the Editorial Policy Checklist.

Statistics

For all statistical analyses, confirm that the following items are present in the figure legend, table legend, main text, or Methods section.

n/a	Confirmed	
☐	☒	The exact sample size (*n*) for each experimental group/condition, given as a discrete number and unit of measurement
☒	☐	A statement on whether measurements were taken from distinct samples or whether the same sample was measured repeatedly
☒	☐	The statistical test(s) used AND whether they are one- or two-sided<br>*Only common tests should be described solely by name; describe more complex techniques in the Methods section.*
☐	☒	A description of all covariates tested
☒	☐	A description of any assumptions or corrections, such as tests of normality and adjustment for multiple comparisons
☐	☒	A full description of the statistical parameters including central tendency (e.g. means) or other basic estimates (e.g. regression coefficient) AND variation (e.g. standard deviation) or associated estimates of uncertainty (e.g. confidence intervals)
☐	☒	For null hypothesis testing, the test statistic (e.g. *F*, *t*, *r*) with confidence intervals, effect sizes, degrees of freedom and *P* value noted<br>*Give P values as exact values whenever suitable.*
☒	☐	For Bayesian analysis, information on the choice of priors and Markov chain Monte Carlo settings
☒	☐	For hierarchical and complex designs, identification of the appropriate level for tests and full reporting of outcomes
☒	☐	Estimates of effect sizes (e.g. Cohen's *d*, Pearson's *r*), indicating how they were calculated

Our web collection on statistics for biologists contains articles on many of the points above.

Software and code

Policy information about availability of computer code

Data collection	No special software was used to collect the data
Data analysis	Mobility data was preprocessed to obtain the stays using the Toyama algorithm, implemented following the details in reference [].<br>Data analysis was done using different R packages. Here is the list of references, included in our Supplementary Information<br>Dowle, M. & Srinivasan, A. data.table: Extension of 'data.frame' (2020). URL https://CRAN.R-project.org/ package=data.table. R package version 1.13.4.<br>[16] LeDell, E. et al. h2o: R Interface for the 'H2O' Scalable Machine Learning Platform (2020). URL https://CRAN. R-project.org/package=h2o. R package version 3.30.0.1.<br>[17] Wickham,H.ggplot2:ElegantGraphicsforDataAnalysis(Springer-VerlagNewYork,2016).URLhttps://ggplot2. tidyverse.org.<br>[18] Cheng, J., Karambelkar, B. & Xie, Y. leaflet: Create Interactive Web Maps with the JavaScript 'Leaflet' Library (2019). URL https://CRAN.R-project.org/package=leaflet. R package version 2.0.3.<br>[19] Walker, K. & Herman, M. tidycensus: Load US Census Boundary and Attribute Data as 'tidyverse' and 'sf'-Ready Data Frames (2020). URL https://CRAN.R-project.org/package=tidycensus. R package version 0.10.2.<br>[20] Walker, K. tigris: Load Census TIGER/Line Shapefiles (2020). URL https://CRAN.R-project.org/package=tigris. R package version 1.0.<br>[21] Hlavac, M. stargazer: Well-Formatted Regression and Summary Statistics Tables. Central European Labour Studies In- stitute (CELSI), Bratislava, Slovakia (2018). URL https://CRAN.R-project.org/package=stargazer. R package version 5.2.2.<br><br>Code to reproduce our results in the figures from the aggregated data is publicly available on github https://github.com/emoro/ Mobility_income_segregation.

For manuscripts utilizing custom algorithms or software that are central to the research but not yet described in published literature, software must be made available to editors and reviewers. We strongly encourage code deposition in a community repository (e.g. GitHub). See the Nature Research guidelines for submitting code & software for further information.

Data

Policy information about availability of data

All manuscripts must include a data availability statement. This statement should provide the following information, where applicable:

- Accession codes, unique identifiers, or web links for publicly available datasets
- A list of figures that have associated raw data
- A description of any restrictions on data availability

The data that support the findings of this study are available from Cuebiq through their Data for Good program, but restrictions apply to the availability of these data, which were used under the licence for the current study and are therefore not publicly available. Information about how to request access to the data and its conditions and limitations can be found in https://www.cuebiq.com/about/data-for-good/. Source anonymized aggregated data to reproduce our results are provided with this paper are publicly available on github: https://github.com/emoro/Mobility_income_segregation.

Other data used comes from the American Community Survey (5-year) from the Census.

Code to reproduce our results in the figures from the aggregated data is publicly available on github https://github.com/emoro/Mobility_income_segregation.

Field-specific reporting

Please select the one below that is the best fit for your research. If you are not sure, read the appropriate sections before making your selection.

☐ Life sciences ☒ Behavioural & social sciences ☐ Ecological, evolutionary & environmental sciences

For a reference copy of the document with all sections, see nature.com/documents/nr-reporting-summary-flat.pdf

Behavioural & social sciences study design

All studies must disclose on these points even when the disclosure is negative.

Study description	Data used are geo-locations from anonymous opted-in devices collected by the company Cuebiq in 11 metro areas in the US. Data has been aggregated at the level of places, categories or census areas where a number of devices are present to prevent de-anonymization. The data is quantitative as it reflects the precise time and geolocation of the anonymous users or metrics from the Census.
Research sample	The sample of users is that described above: anonymous opted-in devices collected by the company Cuebiq.
Sampling strategy	We only selected users for which mobility activity was detected at least in the period studied. See the Methods section for the full detail of our sampling strategy. To minimize the potential bias of geographical penetration of our users, we have implemented post-stratification techniques. All details about our sampling method and post-stratification techniques can be found in the Supplementary Material
Data collection	Data was collected using the geo-location of the users by the Cuebiq company using the location provided by different applications in their mobile phones
Timing	Data was collected from October 2016 through March 2017
Data exclusions	No data was excluded
Non-participation	Only anonymous opted-in devices where used in the analysis
Randomization	The data collected is observational and does not come from an experiment. Thus, this is not applicable.

Reporting for specific materials, systems and methods

We require information from authors about some types of materials, experimental systems and methods used in many studies. Here, indicate whether each material, system or method listed is relevant to your study. If you are not sure if a list item applies to your research, read the appropriate section before selecting a response.

Materials & experimental systems

n/a	Involved in the study
☒	☐ Antibodies
☒	☐ Eukaryotic cell lines
☒	☐ Palaeontology and archaeology
☒	☐ Animals and other organisms
☐	☒ Human research participants
☒	☐ Clinical data
☒	☐ Dual use research of concern

Methods

n/a	Involved in the study
☒	☐ ChIP-seq
☒	☐ Flow cytometry
☒	☐ MRI-based neuroimaging

Human research participants

Policy information about studies involving human research participants

Population characteristics	See above
Recruitment	Data used are geo-locations from anonymous opted-in devices collected by the company Cuebiq in 11 metro areas in the US. Data has been aggregated at the level of places, categories or census areas where a number of devices are present to prevent de-anonymization. We only selected users for which mobility activity was detected at least in the period studied. To minimize the potential bias of geographical penetration of our users, we have implemented post-stratification techniques. All details about our sampling method and post-stratification techniques can be found in the Supplementary Material
Ethics oversight	Since the data used was anonymized and aggregated at the place, category or census areas, we were granted an "Exemption" by the MIT Committee on the Use of Humans as Experimental Subjects (COUHES protocol #1812635935) and its extension #E-2962

Note that full information on the approval of the study protocol must also be provided in the manuscript.
